# Structural identifiability of cyclic graphical models of biological networks with latent variables

**DOI:** 10.1186/s12918-016-0287-y

**Published:** 2016-06-13

**Authors:** Yulin Wang, Na Lu, Hongyu Miao

**Affiliations:** School of Computer Science and Engineering, University of Electronic Science and Technology of China, Chengdu, Sichuan China; State Key Laboratory for Manufacturing Systems Engineering, Systems Engineering Institute, Xi’an Jiaotong University, Xi’an, Shaanxi China; Department of Biostatistics, School of Public Health, University of Texas Health Science Center at Houston, Houston, TX 77030 USA

**Keywords:** Biological network, Graphical model, Structural identifiability analysis, Structural equation model, Symbolic-free elimination

## Abstract

**Background:**

Graphical models have long been used to describe biological networks for a variety of important tasks such as the determination of key biological parameters, and the structure of graphical model ultimately determines whether such unknown parameters can be unambiguously obtained from experimental observations (i.e., the identifiability problem). Limited by resources or technical capacities, complex biological networks are usually partially observed in experiment, which thus introduces latent variables into the corresponding graphical models. A number of previous studies have tackled the parameter identifiability problem for graphical models such as linear structural equation models (SEMs) with or without latent variables. However, the limited resolution and efficiency of existing approaches necessarily calls for further development of novel structural identifiability analysis algorithms.

**Results:**

An efficient structural identifiability analysis algorithm is developed in this study for a broad range of network structures. The proposed method adopts the Wright’s path coefficient method to generate identifiability equations in forms of symbolic polynomials, and then converts these symbolic equations to binary matrices (called identifiability matrix). Several matrix operations are introduced for identifiability matrix reduction with system equivalency maintained. Based on the reduced identifiability matrices, the structural identifiability of each parameter is determined. A number of benchmark models are used to verify the validity of the proposed approach. Finally, the network module for influenza A virus replication is employed as a real example to illustrate the application of the proposed approach in practice.

**Conclusions:**

The proposed approach can deal with cyclic networks with latent variables. The key advantage is that it intentionally avoids symbolic computation and is thus highly efficient. Also, this method is capable of determining the identifiability of each single parameter and is thus of higher resolution in comparison with many existing approaches. Overall, this study provides a basis for systematic examination and refinement of graphical models of biological networks from the identifiability point of view, and it has a significant potential to be extended to more complex network structures or high-dimensional systems.

**Electronic supplementary material:**

The online version of this article (doi:10.1186/s12918-016-0287-y) contains supplementary material, which is available to authorized users.

## Background

Although the reductionism approaches have led to tremendous success in advancing our knowledge and understanding of individual biological components and their functions, it has been broadly recognized that many organic/cellular functions or disorders cannot be attributed to an individual molecule [1]. Instead, numerous biological components interact with each other and orchestrate various dynamic events that are critical to the beginning and extension of life [2]. To systematically investigate and understand such complex interactions, a variety of biological networks (e.g., transcriptional and post-transcriptional regulatory networks [3–6], functional RNA networks [7–9], protein-protein interaction networks [10, 11], and metabolic networks [12, 13]) have necessarily been constructed based on experimental observations or predictions. Nowadays, biological networks are playing critical roles in biomedical research and practice at multiple levels or scales (e.g., genetics [14], immunology [15], cancer [16], drug discovery [17, 18]), and the associated modeling and computation techniques and tools are under active development for network property investigation, network structure identification, experimental data analysis and interpretation, and so on [1, 15–19].

Graphical models are one of the most powerful mathematical languages for biological network representation, and have long been used for various quantitative analysis tasks [19–21]. In particular, the determination of unknown model parameter values from experimental data is of fundamental importance to many other critical tasks (e.g., computer simulation or prediction, network structure refinement), and it should be stressed that parameter identifiability is one of the first questions that needs to be answered before any statistical method can be applied to obtain accurate and reliable estimates of unknown parameters [20]. More specifically, limited by resources or technical capabilities, it is not uncommon that only part of the nodes or interactions (i.e., edges) in a biological network can be experimentally observed such that the values of certain unknown parameters associated with those unobserved nodes or edges cannot be uniquely determined from experimental data due to the lack of information. However, even if all the nodes and edges are observed, identifiability issues may still occur due to, e.g., model misspecification. It is thus necessary to develop identifiability analysis techniques for graphical models with or without latent variables.

Since graphical models refer to a broad range of mathematical formulations [19–22], it is impossible to explore the identifiability analysis techniques for all different types of graphical models in one study. Here we focus on the structural identifiability analysis problem of static linear structural equation model (SEM), which is a representative and generic graphical model type that has been widely used in many different research areas such as clinical psychology, education, cognitive science, behavioral medicine, developmental psychology, casual inference [23, 24], and systems biology [25–27]. A number of previous studies have proposed identifiability analysis techniques for linear SEMs with or without latent variables [23, 24, 28–43]. More specifically, the traditional method described in [23] constructs a so-called system matrix from a given model structure and derives the rank and order conditions based on this matrix for identifiability analysis. However, this approach can only handle comparatively simple network structures (e.g., block recursive models [23]) without latent variables, and cannot deal with the disturbance correlation between variables (i.e., nodes). To deal with a broader range of model structures, investigators from different disciplines have made further attempts by considering the topological or other features of certain networks. For instance, several previous studies have derived the sufficient criteria for parameter identifiability based on local characteristics of subnetworks, including Pearl’s back door and front door criteria [24], Brito and Pearl’s generalized instrumental variable criterion [30], and Tian’s accessory set approach [41]. For certain network structures, sufficient conditions for parameter identifiability have also been established for the entire network instead of subnetworks; e.g., Brito and Pearl’s conditions for bow-free models [28], Brito and Pearl’s auxiliary sets condition for directed acyclic graph (DAG) models [36], Drton’s condition based on injective parametrization of mixed graphs [35], and Foygel’s half-trek criterion for mixed graphs [37]. While the criteria and conditions mentioned above are important progresses made in the field, they only provide a partial or overall assessment of parameter identifiability. To determine the identifiability of every single parameter in the model, Tian [32] adopted the partial regression analysis technique, but this approach can only handle a special class of P-structure-free SEMs. Also, Sullivant et al. [34] tackled this problem using a computer algebra method, which turns out to be applicable only to SEMs with a small number of variables due to the prohibitive computation costs associated with Gröbner basis reduction. Therefore, it is still necessary to develop more efficient single-parameter-level approaches for structural identifiability analysis of whole networks.

In this study, we developed a novel and efficient approach for structural identifiability analysis of cyclic linear SEMs with latent variables. The proposed method is applicable to both directed cyclic and acyclic graphs with or without latent variables, and thus presents an extension of existing algorithms in terms of generality. Different from other existing algebraic approaches, although our method uses the Wright’s path coefficient method to generate identifiability equations in forms of nonlinear symbolic polynomials, it avoids the expensive symbolic computations (e.g., Gröbner basis reduction) by converting identifiability equations to binary matrices, and is thus highly efficient. Moreover, in contrast to other methods that can only draw conclusions on the overall identifiability of a model, the proposed method can determine the identifiability of each single unknown parameter, and is thus of higher resolution and enables researchers to locate the problematic subnetwork structures to refine model structures or improve experimental design. We collected a number of benchmark models from literature and verified the validity of our method using those models. Finally, we applied our method to the network module for influenza A virus (IAV) within-host replication to gain insights into parameter identifiability and experimental design.

## Methods

The key definitions and steps involved in the proposed algorithm are described in this section, including the definition of structural identifiability analysis for cyclic SEMs, the generation of identifiability equations, the conversion to identifiability matrices, and the symbolic-free identifiability determination based on the reduced identifiability matrices. The necessary theoretical justification is also given.

### SEM and structural identifiability

The structural equation models considered in this study correspond to a mixed cyclic graph G = (V, D, U), where V is a set of vertices, D a set of directed edges, and U a set of undirected edges. That is, in the SEM, each model variable *Y*_*i*_ corresponds to a vertex *V*_*i*_ (*i* = 1, 2, …, *n*), the structure of the coefficient matrix **C** = [*c*_*ij*_] is specified by D (i.e., *c*_*ij*_ exists if a directed edge from *V*_*j*_ to *V*_*i*_ is in D; otherwise, *c*_*ij*_ = 0 if no edge exists in D from *V*_*j*_ to *V*_*i*_, *i* ≠ *j*), and the existence of disturbance correlation between two variables is given by U. Here disturbance refers to all the omitted causes of a variable, and disturbance correlation is the correlation between two variables due to the existence of common omitted cause(s) shared by the two variables [24]. As suggested in a number of studies [24, 28–30, 32, 34, 35, 37, 44], it is not always necessary to classify the model variables into endogenous or exogenous; therefore, following the notation in Drton et al. [35], the SEM representation of a cyclic graph can be given as follows

1 $$ {Y}_i={\displaystyle \sum_{j\in Parent(i)}}{c}_{ij}{Y}_j+{\varepsilon}_i,\kern2.5em i,j=1,\cdots, n, $$

where *c*_*ij*_ denotes the weight of the directed edge *V*_*j*_ → *V*_*i*_, *ε*_*i*_ denotes the random error that follows a certain distribution (Gaussian or non-Gaussian [31, 38]) with mean zero, and *Parent*(*i*) denotes the set of parent nodes of node *i*. Without loss of generality, all ${Y}_i$ s are assumed to be standardized via necessary transform [45]. To distinguish observed variables from latent variables, the superscripts *o* and *l* can be used (i.e., *Y*_*i*_^*o*^ and *Y*_*i*_^*l*^). Furthermore, let *σ*_*ij*_ = Cov(*Y*_*i*_, *Y*_*j*_) denote the covariance between two node variables. Also, let *ω*_*ij*_ denote the disturbance correlation between *Y*_*i*_ and *Y*_*j*_; by definition, *ω*_*ij*_ = 0 if no undirected edge *V*_*j*_ ↔ *V*_*i*_ can be found in U. For convenience, we denote the covariance matrix and the disturbance correlation matrix as **Σ** = [*σ*_*ij*_] and **Ω** = [*ω*_*ij*_], respectively.

In general, the purpose of identifiability analysis is to verify whether certain unknown parameters can be uniquely and reliably determined for given model structures with or without considering data noise or model uncertainty [24, 28–30, 32, 34, 35, 37, 44]. Here the goal of structural identifiability analysis of SEMs is to determine whether the unknown parameters in matrices **C** and **Ω** can be unambiguously determined for a given network structure G = (V, D, U). This type of analysis does not take specific data distribution or noise level into consideration as its primary concern is not the robustness but the accuracy of parameter estimation via examining possible flaws in model structure or experimental design. More importantly, the structural identifiability of a parameter can be verified by checking its number of solutions to a system of polynomial equations. That is, a parameter is globally identifiable if only one solution exists, locally identifiable if a finite number of solutions exist, and unidentifiable if an infinite number of solutions exist [20].

**Fig. 1:**
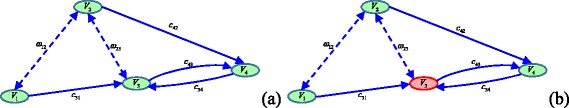
A mixed graph example, where the dashed bidirectional arrow represents the disturbance correlation between the two variables. **a** Without latent variables; **b** With latent variable ${Y}_3$ (labelled in red)

For illustration purpose, we consider the mixed graph example in Fig. 1. The corresponding linear SEM is given as follows:

2 $$ \left\{\begin{array}{l}{Y}_1={\varepsilon}_1\\ {}{Y}_2={\varepsilon}_2\\ {}{Y}_3={c}_{31}{Y}_1+{c}_{34}{Y}_4+{\varepsilon}_3\\ {}{Y}_4={c}_{42}{Y}_2+{c}_{43}{Y}_3+{\varepsilon}_4\\ {}{\omega}_{12}\ne 0\\ {}{\omega}_{23}\ne 0\end{array}\right., $$

the coefficient and the disturbance correlation matrices are

3 $$ \mathbf{C}=\left[\begin{array}{cccc}\hfill 0\hfill & \hfill 0\hfill & \hfill 0\hfill & \hfill 0\hfill \\ {}\hfill 0\hfill & \hfill 0\hfill & \hfill 0\hfill & \hfill 0\hfill \\ {}\hfill {c}_{31}\hfill & \hfill 0\hfill & \hfill 0\hfill & \hfill {c}_{34}\hfill \\ {}\hfill 0\hfill & \hfill {c}_{42}\hfill & \hfill {c}_{43}\hfill & \hfill 0\hfill \end{array}\right]\kern0.5em \mathrm{and}\kern0.5em \boldsymbol{\Omega} =\left[\begin{array}{cccc}\hfill 0\hfill & \hfill {\omega}_{12}\hfill & \hfill 0\hfill & \hfill 0\hfill \\ {}\hfill {\omega}_{12}\hfill & \hfill 0\hfill & \hfill {\omega}_{23}\hfill & \hfill 0\hfill \\ {}\hfill 0\hfill & \hfill {\omega}_{23}\hfill & \hfill 0\hfill & \hfill 0\hfill \\ {}\hfill 0\hfill & \hfill 0\hfill & \hfill 0\hfill & \hfill 0\hfill \end{array}\right], $$

respectively, and the covariance matrices for Fig. 1a, b are

4 $$ {\boldsymbol{\Sigma}}_a=\left[\begin{array}{cccc}\hfill {\sigma}_{11}\hfill & \hfill {\sigma}_{12}\hfill & \hfill {\sigma}_{13}\hfill & \hfill {\sigma}_{14}\hfill \\ {}\hfill {\sigma}_{12}\hfill & \hfill {\sigma}_{22}\hfill & \hfill {\sigma}_{23}\hfill & \hfill {\sigma}_{24}\hfill \\ {}\hfill {\sigma}_{13}\hfill & \hfill {\sigma}_{23}\hfill & \hfill {\sigma}_{33}\hfill & \hfill {\sigma}_{34}\hfill \\ {}\hfill {\sigma}_{14}\hfill & \hfill {\sigma}_{24}\hfill & \hfill {\sigma}_{34}\hfill & \hfill {\sigma}_{44}\hfill \end{array}\right]\kern0.5em \mathrm{and}\kern0.5em {\boldsymbol{\Sigma}}_b=\left[\begin{array}{cccc}\hfill {\sigma}_{11}\hfill & \hfill {\sigma}_{12}\hfill & \hfill -\hfill & \hfill {\sigma}_{14}\hfill \\ {}\hfill {\sigma}_{12}\hfill & \hfill {\sigma}_{22}\hfill & \hfill -\hfill & \hfill {\sigma}_{24}\hfill \\ {}\hfill -\hfill & \hfill -\hfill & \hfill -\hfill & \hfill -\hfill \\ {}\hfill {\sigma}_{14}\hfill & \hfill {\sigma}_{24}\hfill & \hfill -\hfill & \hfill {\sigma}_{44}\hfill \end{array}\right], $$

respectively, where the symbol “—” denotes unknown covariance due to the existence of the latent variable *Y*_3_. For the model corresponding to Fig. 1b, the structure identifiability problem is to determine the number of solutions of each unknown parameter in matrices C and Ω (i.e., *c*_31_, *c*_34_, *c*_42_, *c*_43_, *ω*_12_ and *ω*_23_).

### Generating identifiability equations

Identifiability equations are obtained after eliminating all latent variables so they are a set of equations that contains only observed variables, unknown parameters and maybe other constants. It has been shown that under the assumption of normally-distributed disturbance, the covariance matrix **Σ** can be expressed in terms of **C** and **Ω**

5 $$ \boldsymbol{\Sigma} ={\left(\mathbf{I}-\mathbf{C}\right)}^{-\mathrm{T}}\boldsymbol{\Omega} {\left(\mathbf{I}-\mathbf{C}\right)}^{-1}, $$

where **I** denotes the identity matrix. If the unknown covariance(s) in **Σ** can be eliminated, Eq. (5) will become a set of equations that involve only the unknown parameters in **C** and **Ω**, and thus has been used as identifiability equations in previous studies [23, 34]. However, this approach needs to calculate the symbolic inversion of the matrix (**I** − **C**) such that it can only handle small models with a few unknown parameters even if with the use of the computer algebra tools [34]. Therefore, here we consider the Wright’s method of path coefficients to generate identifiability equations [45, 46]. Briefly, the Wright’s method considers the fact that two node variables are correlated with each other if there exists a path between these two nodes in a given network structure, and thus calculate the covariance between two node variables by adding the products of edge coefficients along each path. This approach can easily generate the identifiability equations in forms of nonlinear symbolic polynomials and has been previously verified and used for identifiability analysis of SEMs [29, 30].

More specifically, for an acyclic linear SEM (also called recursive SEM that corresponds to a directed acyclic graph), the covariance *σ*_*ij*_ of a pair of variables *Y*_*i*_ and *Y*_*j*_ is calculated as ${\sigma}_{ij}={\displaystyle \sum_{pat{h}_k}}{\displaystyle \prod_{edg{e}_l}}{\theta}_l$, where *θ*_*l*_ is the coefficient of the *l*-th edge in path *k* (i.e., *c*_*pq*_ or *ω*_*pq*_associated with a directed edge *V*_*q*_ → *V*_*p*_ or an undirected *V*_*q*_ ↔ *V*_*p*_). Note that each path includes at most one undirected edge and must be unblocked [29, 30, 45, 46] (i.e., the two end nodes of a path are connected in the directed graph part G = (V, D)). For a cyclic linear SEM (also called non-recursive), the directed graph part G = (V, D) contains one or multiple cycles such that we need to enumerate all distinct cycles and paths. The key issue is that, for two nodes in the same cycle, there are two different sets of paths *V*_*i*_ → ⋯ → *V*_*j*_and *V*_*j*_ → ⋯ → *V*_*i*_ according to the Wright’s method. That is, two different sets of equations can be generated for *σ*_*ij*_ and *σ*_*ji*_, respectively, although *σ*_*ij*_ = *σ*_*ji*_. Furthermore, for any latent variable *Y*_*i*_ in a SEM, the covariance between *Y*_*i*_ and any other variable is unknown and cannot be used to generate identifiability equations (see **Σ**_*b*_, the corresponding covariance matrix of Fig. 1b). In short, the existence of cycles or latent variables will lead to the increase or decrease of the number of identifiability equations, respectively, and thus will eventually affect the number of solutions of unknown model parameters.

Back to the examples in Fig. 1, it can be shown that the identifiability equations generated using the Wright’s method for Fig. 1a, b are

6 $$ \left\{\begin{array}{l}{\sigma}_{12}={\omega}_{12}+{c}_{31}{\omega}_{23}\\ {}{\sigma}_{13}={c}_{31}+{\omega}_{12}{c}_{42}{c}_{34}\\ {}{\sigma}_{14}={c}_{31}{c}_{43}+{\omega}_{12}{c}_{42}+{c}_{31}{\omega}_{23}{c}_{42}\\ {}{\sigma}_{23}={c}_{42}{c}_{34}+{\omega}_{23}+{\omega}_{12}{c}_{31}\\ {}{\sigma}_{24}={c}_{42}+{\omega}_{23}{c}_{43}+{\omega}_{12}{c}_{31}{c}_{43}\\ {}{\sigma}_{34}={c}_{43}+{\omega}_{23}{c}_{42}\\ {}{\sigma}_{34} = {c}_{34}\end{array}\right., $$

and

7 $$ \left\{\begin{array}{l}{\sigma}_{12}={\omega}_{12}+{c}_{31}{\omega}_{23}\\ {}{\sigma}_{14}={c}_{31}{c}_{43}+{\omega}_{12}{c}_{42}+{c}_{31}{\omega}_{23}{c}_{42}\\ {}{\sigma}_{24}={c}_{42}+{\omega}_{23}{c}_{43}+{\omega}_{12}{c}_{31}{c}_{43}\end{array}\right., $$

respectively. In Fig. 1a, because the two nodes *V*_3_ and *V*_4_ are in the same cycle, we have *σ*_34_ = *c*_43_ + *ω*_23_*c*_42_ for *V*_3_ → *V*_4_ and *σ*_43_ = *c*_34_ for *V*_4_ → *V*_3_ in Eq. (6). In Fig. 1b, since the node *V*_3_ is unobserved, the covariance *σ*_13_, *σ*_23_ and *σ*_34_ are unavailable for identifiability analysis as shown in Eq. (7).

### Generating identifiability matrices

The identifiability equations are symbolic polynomials and are nonlinear with respect to unknown parameters. Simplifying and solving such equations using the computer algebra algorithms usually presents significant computational challenges [34]. Here we propose a novel and efficient approach, and the basic idea is to convert the identifiability equations to binary matrices, called identifiability matrices.

For each identifiability equations, one identifiability matrix is generated. More specifically, each column of the matrix corresponds to an unknown parameter, and each row corresponds to a monomial ${\displaystyle \prod_{edg{e}_l\ }}{\theta}_l$. If the *i*-th monomial of an identifiability equation contains the *j*-th unknown parameter, then the corresponding matrix element *m*_*ij*_ is equal to 1, otherwise *m*_*ij*_ = 0. Note that when generating the identifiability matrices, constant terms or known coefficients are not considered since they have no effects on the identifiability of unknown parameters. For illustration purpose, the list of identifiability matrices generated from Eq. (6) is given as follows

$$ {c}_{31}\ {c}_{34}\ {c}_{42}\ {c}_{43}\ {\omega}_{12}\ {\omega}_{23} $$

$$ {\sigma}_{12}\left[\begin{array}{cccccc}\hfill 0\hfill & \hfill 0\hfill & \hfill 0\hfill & \hfill 0\hfill & \hfill 1\hfill & \hfill 0\hfill \\ {}\hfill 1\hfill & \hfill 0\hfill & \hfill 0\hfill & \hfill 0\hfill & \hfill 0\hfill & \hfill 1\hfill \end{array}\right], $$

$$ {\sigma}_{13}\left[\begin{array}{cccccc}\hfill 1\hfill & \hfill 0\hfill & \hfill 0\hfill & \hfill 0\hfill & \hfill 0\hfill & \hfill 0\hfill \\ {}\hfill 0\hfill & \hfill 1\hfill & \hfill 1\hfill & \hfill 0\hfill & \hfill 1\hfill & \hfill 0\hfill \end{array}\right], $$

$$ {\sigma}_{14}\left[\begin{array}{cccccc}\hfill 1\hfill & \hfill 0\hfill & \hfill 0\hfill & \hfill 1\hfill & \hfill 0\hfill & \hfill 0\hfill \\ {}\hfill 0\hfill & \hfill 0\hfill & \hfill 1\hfill & \hfill 0\hfill & \hfill 1\hfill & \hfill 0\hfill \\ {}\hfill 1\hfill & \hfill 0\hfill & \hfill 1\hfill & \hfill 0\hfill & \hfill 0\hfill & \hfill 1\hfill \end{array}\right], $$

$$ {\sigma}_{23}\left[\begin{array}{cccccc}\hfill 0\hfill & \hfill 1\hfill & \hfill 1\hfill & \hfill 0\hfill & \hfill 0\hfill & \hfill 0\hfill \\ {}\hfill 0\hfill & \hfill 0\hfill & \hfill 0\hfill & \hfill 0\hfill & \hfill 0\hfill & \hfill 1\hfill \\ {}\hfill 1\hfill & \hfill 0\hfill & \hfill 0\hfill & \hfill 0\hfill & \hfill 1\hfill & \hfill 0\hfill \end{array}\right], $$

$$ {\sigma}_{24}\left[\begin{array}{cccccc}\hfill 0\hfill & \hfill 0\hfill & \hfill 1\hfill & \hfill 0\hfill & \hfill 0\hfill & \hfill 0\hfill \\ {}\hfill 0\hfill & \hfill 0\hfill & \hfill 0\hfill & \hfill 1\hfill & \hfill 0\hfill & \hfill 1\hfill \\ {}\hfill 1\hfill & \hfill 0\hfill & \hfill 0\hfill & \hfill 1\hfill & \hfill 1\hfill & \hfill 0\hfill \end{array}\right], $$

$$ {\sigma}_{34}\left[\begin{array}{cccccc}\hfill 0\hfill & \hfill 0\hfill & \hfill 0\hfill & \hfill 1\hfill & \hfill 0\hfill & \hfill 0\hfill \\ {}\hfill 0\hfill & \hfill 0\hfill & \hfill 1\hfill & \hfill 0\hfill & \hfill 0\hfill & \hfill 1\hfill \end{array}\right], $$

$$ {\sigma}_{34}\left[\begin{array}{cccccc}\hfill 0\hfill & \hfill 1\hfill & \hfill 0\hfill & \hfill 0\hfill & \hfill 0\hfill & \hfill 0\hfill \end{array}\right]. $$

From Eq. (7), we can generate three matrices for *σ*_12_, *σ*_14_ and *σ*_24_, respectively, which are the same as those from Eq. (6) and thus not shown here.

### Reducing identifiability matrices

If all elements are 0 in an identifiability matrix **M**, it is simply a zero matrix (denoted by **M**_*Z*_). Such matrices may occur during the reduction process. However, a zero matrix is not useful to identifiability analysis because it contains no unknown parameters. Therefore, once an identifiability matrix becomes a zero matrix after a certain number of reduction operations, it can be removed. For the same reason, a zero row in an identifiability matrix can also be deleted.

Given an identifiability matrix **M** with a row number *N*_*R*_(**M**) greater than 1, if all the rows in **M** are the same, such a matrix is called a repeated matrix (denoted by **M**_*R*_). The corresponding identifiability equation of a repeated matrix is ${\sigma}_{ij}={a}_1{\displaystyle \prod_l}{\theta}_l+{a}_2{\displaystyle \prod_l}{\theta}_l\cdots +{a}_K{\displaystyle \prod_l}{\theta}_l$, where all the monomials are the same except for the constant coefficients {*a*_1_, *a*_2_, …, *a*_*K*_} in the front. Since the equation can be simplified to ${\sigma}_{ij}=A\cdot {\displaystyle \prod_l}{\theta}_l$, where *A* = *a*_1_ + *a*_2_ + ⋯ + *a*_*K*_, the repeated identifiability matrix can be replaced by a single row without loss of information (denoted by **M**_*RI*_).

**Fig. 2:**
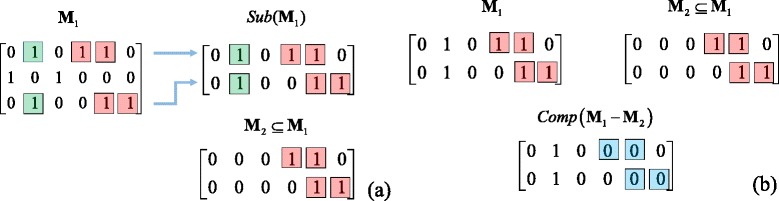
Illustration of **a** sub-matrix and matrix inclusion; and **b** complement matrix. Different colors are used to highlight the elements that remain the same or become different in different matrices

Further notations are needed to describe the relationships between two identifiability matrices. First, if all the rows in matrix **M**_2_ are from another matrix **M**_1_, **M**_2_ is called a sub-matrix of **M**_1_, denoted by **M**_2_ = *Sub*(**M**_1_), and the remaining part is denoted by *Rem*(**M**_1 − 2_). Second, for two identifiability matrix **M**_1_ and **M**_2_ (*N*_*R*_(**M**_1_) ≥ *N*_*R*_(**M**_2_)), if a sub-matrix of **M**_1_, denoted by **M**_3_, can be found such that it has the same number of rows as **M**_2_, and every element “1” in **M**_2_ is also a “1” in **M**_3_, then we call **M**_1_ includes **M**_2_, denoted by **M**_2_ ⊆ **M**_1_. An example of such a relationship is given in Fig. 2a for illustration purpose. Third, given two identifiability matrices **M**_1_ and **M**_2_ such that *N*_*R*_(**M**_1_) = *N*_*R*_(**M**_2_) and **M**_2_ ⊆ **M**_1_, then **M**_3_ = (**M**_1_ − **M**_2_) is called a complement matrix, denoted by *Comp*(**M**_1_ − **M**_2_). See Fig. 2b for illustration of the complement matrix concept.

**Fig. 3:**
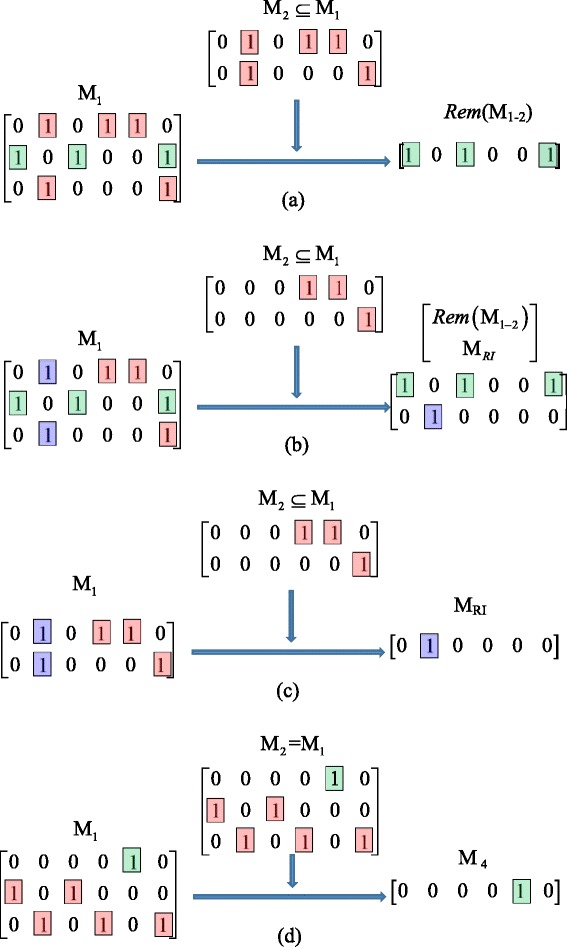
Several examples of the row deletion operation. Different colors are used to highlight the elements that remain the same or become different in different matrices. (**a**) Case 1 of reducing M_1_ by M_2_ ; (**b**) Case 2 of reducing M_1_ by M_2_ ; (**c**) Case 3 of reducing M_1_ by M_2_ ; (**d**) Case 4 of reducing M_1_ by M_2_

Now the key issue is that the identifiability matrices before and after reduction should be equivalent; that is, the two sets of matrices should lead to the same conclusions on parameter identifiability. Let **M**_1_ ~ **M**_2_ denote two equivalent matrices, here we show that the following operations for matrix reduction can meet the requirement of identifiability equivalency (see Additional file 1 for theoretical justification):

i) **Row swap**. Let ***R***_*i*_ and ***R***_*j*_ (*i* ≠ *j*) denote two different rows of an identifiability matrix **M**_1_, and let **M**_2_ denote the matrix generated after swapping ***R***_*i*_ and ***R***_*j*_, then **M**_1_ ~ **M**_2_.
ii) **Redundant row removal**. Let ***R***_*i*_ and ***R***_*j*_ (*i* ≠ *j*) denote two different rows of an identifiability matrix **M**_1_. If ***R***_*i*_ = ***R***_*j*_ and let **M**_2_ denote the matrix generated after removing ***R***_*i*_ or ***R***_*j*_, then **M**_1_ ~ **M**_2_.
iii) **Row deletion**. Let **M**_1_ and **M**_2_ be two identifiability matrices, which correspond to two different identifiability equations, such that *N*_*R*_(**M**_1_) > 1 and **M**_2_ ⊆ **M**_1_. Also, let **M**_3_ = *sub*(**M**_1_) be a submatrix consisting of **M**_1_’s rows that **M**_2_ has in **M**_1_. See Fig. 3 for examples.

  If *Rem*(**M**_1 − 2_) ≠ **M**_*Z*_ and *Comp*(**M**_3_ − **M**_2_) = **M**_*Z*_, then **M**_1_ can be reduced to *Rem*(**M**_1 − 2_) without altering the parameter identifiability;
  If *Rem*(**M**_1 − 2_) ≠ **M**_*Z*_ and *Comp*(**M**_3_ − **M**_2_) = **M**_*R*_, then **M**_1_ can be reduced to $$ \left[\begin{array}{c}\hfill Rem\left({\mathbf{M}}_{1-2}\right)\hfill \\ {}\hfill {M}_{RI}\hfill \end{array}\right] $$ without altering the parameter identifiability;
  If *Rem*(**M**_1 − 2_) = **M**_*Z*_ and *Comp*(**M**_3_ − **M**_2_) = **M**_*R*_, then **M**_1_ can be reduced to **M**_*RI*_ without altering the parameter identifiability;
  If *Rem*(**M**_1 − 2_) = **M**_*Z*_ and *Comp*(**M**_3_ − **M**_2_) = **M**_*z*_ (i.e., **M**_1_ = **M**_2_ = **M**_3_), and take the row which has the least “1” elements in **M**_1_ to form a new matrix **M**_4_, then **M**_1_ can be reduced to **M**_4_ without altering the parameter identifiability.

**Fig. 4:**
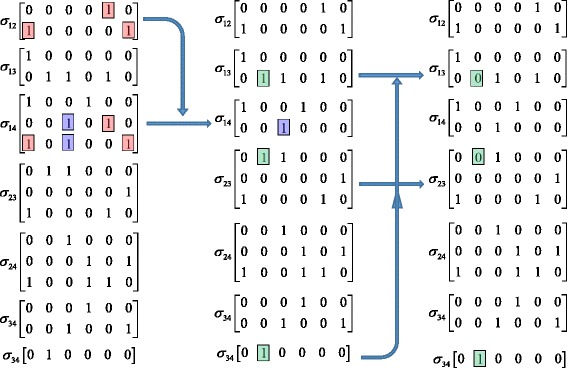
The reduction process of the identifiability matrices from Fig. 1a. In the left column, we subtract matrix *σ*_12_ from matrix *σ*_14_; in the middle column, we then subtract matrix *σ*_34_ from matrices *σ*_13_ and *σ*_23_; and finally, we get the reduction result in the right column

The reduction process is iterative, and it stops until we cannot reduce the identifiability matrices further more. For illustration purpose, the whole reduction process for the identifiability matrices from Fig. 1a is shown in Fig. 4. The computation complexity of the reduction process depends on the number of rows in the identifiability matrices (denoted by *m*). In the worst scenario where every pair of rows need to be compared, the computing cost is O(*m*^2^); however, the efficiency can be improved if all the rows can be sorted before row comparison according to the positions of the “1” elements from left to right.

### Determining parameter identifiability

After all identifiability matrices are reduced to the simplest forms using the operations described in the previous section, the identifiability of all the unknown parameters can be determined. The simplest case is to find out the globally identifiable, That is, if a matrix has only one row and this row has only one “1” element, the parameter corresponding to that “1” element is then globally identifiable, because the associated identifiability equation is in the form *θ*_*i*_ = const. For example, the matrix of the bottom *σ*_34_ matrix in Fig. 4 has only one row with only one “1” element, so the parameter *c*_34_ corresponding to the “1” element is globally identifiable.

**Fig. 5:**
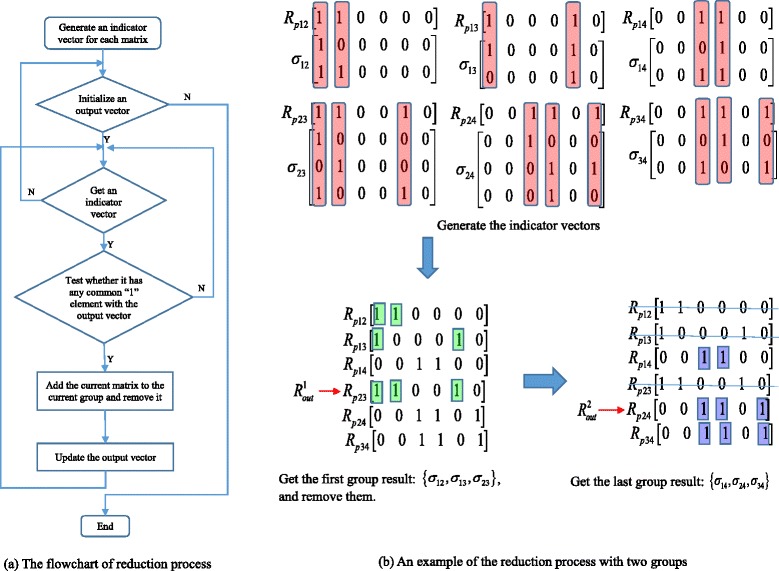
Illustration of the grouping algorithm. **a** The reduction process flowchart; **b** An example of the reduction process with two matrix groups

After removing all the matrices for globally identifiable parameters, the remaining matrices all have more than one “1” elements and they need to be regrouped and decoupled. That is, if the *i*-th columns of matrices **M**_1_ and **M**_2_ both contain one or more “1” elements, **M**_1_ and **M**_2_ will be in the same group. Here we describe the algorithm for grouping the identifiability matrices (see Fig. 5 for illustration).

(i) Apply the bit-OR operation to the first two rows, and then to the result and the 3rd row, and so on until the last row of a matrix to generate an indicator vector ***R***_*p*_ such that each “1” element in this vector indicates the existence of a certain parameter;
(ii) Initialize an output vector ***R***_*out*_ as the vector ***R***_*p*_ that contains largest number of “1” elements among all ***R***_*p*_ s;
(iii) Check each of the ***R***_*p*_ vectors to verify whether it has any common “1” element with ***R***_*out*_ using the bit-AND operation. If the bit-AND result is not a zero vector, then the identifiability matrix corresponding to ***R***_*p*_ will be added to the current group. Then update ***R***_*out*_ by applying the bit-OR operation to ***R***_*out*_ and the bit-AND result;
(iv) Repeat Step (iii) until no more matrices can be added to the current group;
(v) Remove all the matrices of the current group, and repeat steps (ii) to (iv) until all different groups are found.

The identifiability of all the parameters in the same group are determined together. According to the definition of identifiability matrix, one can tell that all the matrices of the same group correspond to a system of coupled polynomial equations, and the critical issue here is to determine the number of solutions of each parameter to these equations. Garcia and Li [47] have theoretically investigated this problem and shown that for a system of *n* polynomial equations with *n* complex variables, the number of solutions is equal to $q={\displaystyle \prod_{i=1}^n}{q}_i$, where *q*_*i*_ is the degree (the power of the highest ordered term) of equation *i*. Therefore, every unknown variable of the system has a unique solution when *q* = 1, and has multiple solutions if *q* > 1. Based on the work of Garcia and Li, we establish the theoretical connection between parameter identifiability and the grouped identifiability matrices, and the theoretical proof is given in Additional file 2 for interested readers.

#### Theorem 1

For the reduced identifiability matrices in the same group, let *N*_*M*_ denote the number of matrices, let *N*_*P*_ denote the number of unknown parameters, and let *N*_max_ be the maximum number of the “1” elements in one row of all the matrices.

- When *N*_*P*_ > *N*_*M*_, all the parameters in the same group are unidentifiable;
- When *N*_*P*_ = *N*_*M*_, the parameters are globally identifiable if *N*_max_ = 1, and locally identifiable if *N*_max_ > 1;
- When *N*_*P*_ < *N*_*M*_, the parameters are at least locally identifiable.

Based on Theorem 1, we can determine the structural identifiability of each parameter for the models in Fig. 1. That is, for the model in Fig. 1a, one can tell that the parameter *c*_34_ is globally identifiable. The remaining matrices are of the same group; and the number of matrices is *N*_*M*_ = 6, the number of unknown parameters is *N*_*P*_ = 5, and *N*_max_ = 5 is greater than 1. Therefore, all the unknown parameters {*c*_31_, *c*_42_, *c*_43_, *ω*_12_, *ω*_23_} are locally identifiable. Similarly for the model in Fig. 1 bone can tell *N*_*M*_ = 3 and *N*_*P*_ = 5 so all the parameters {*c*_31_, *c*_34_, *c*_42_, *c*_43_, *ω*_12_, *ω*_23_} are unidentifiable.

## Results and discussion

### Overview of the framework

Graphical models have long been used to describe biological networks for a variety of important tasks like network structure identification. Many such quantitative analyses involve determination of unknown model parameters from experimental data, and identifiability analysis is a necessary step to perform before parameter estimation to assure the accuracy or robustness of the estimates. In particular, structural identifiability analysis can help to locate mis-specified substructures of models or improve experimental design with considering unobserved variables. A number of previous studies have proposed identifiability analysis techniques for structural equations models, with particular attention paid to specific network structures (e.g., directed acyclic graphs) or experimental conditions (e.g., without latent variables). Also, existing methods usually give an overall assessment instead of verifying the identifiability of each single parameter, and the use of symbolic computation algorithms (e.g., Gröbner basis reduction) is computationally expensive and has significantly limited the applications of these methods in more complex biological network structures and moderate to high-dimensional systems.

**Fig. 6:**
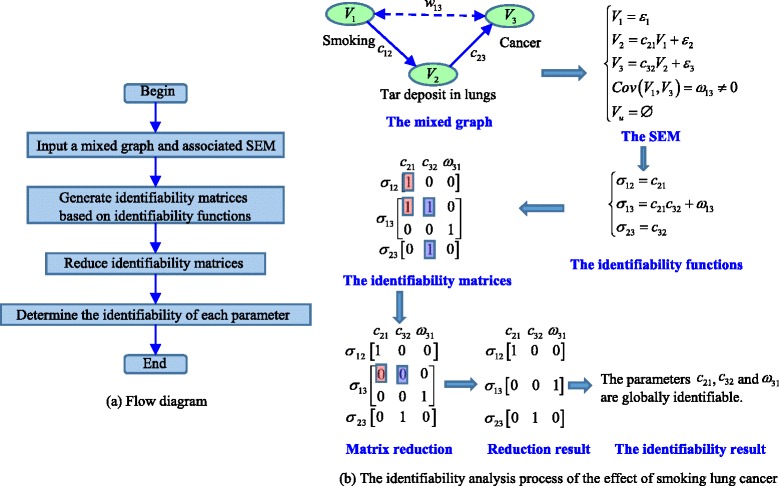
Illustration of the whole process of identifiability analysis: **a** Flowchart; **b** A simple example

In this study, we develop a novel and efficient structural identifiability analysis technique to deal with a broader range of biological networks. To the best knowledge of the authors, the proposed method makes several worthwhile progresses in comparison with the previous work. First, the covariance between two observed variables can always be calculated (e.g., sample covariance) and thus treated as known, and a symbolic equation can be generated for this covariance by considering the effects of one variable on the other propagating through the path(s) between the two nodes. We adopt the Wright’s path coefficient method [45, 46] for identifiability equation generation, which is not only more efficient than the approach of symbolic matrix inversion [34] but also can deal with cyclic networks with latent variables. Second, the computer algebra algorithms nowadays are only capable of efficiently solving nonlinear symbolic equations with a small number of variables, we propose a novel strategy to convert each symbolic equation to an identifiability matrix, and we also develop the necessary operations (e.g., row deletion) for identifiability matrix reduction without jeopardizing the equivalency of the identifiability results. Third, we present a strategy for regrouping the reduced identifiability matrices, and provide the guidelines with theoretical justification for determining parameter identifiability from the grouped matrices. The several contributions described above are in the same order of the algorithm pipeline, as depicted in Fig. 6. Finally, it should be stressed that the proposed algorithm is highly efficient because the main operations involved here are simple matrix manipulations like logical bitwise operations or matrix row deletion. For instance, it will take 0.3 to 4.5 s on a modern desktop computer to obtain the identifiability analysis results for a SEM with 4 nodes, 3 directed edges and 3 undirected edges using the computer algebra method [34]; however, it will only take several milliseconds or less to reach the conclusions using the method proposed in this study as binary matrix operations are extremely efficient. It should be mentioned that many existing methods cannot be directly compared with the proposed method because they are not designed for static SEMs or they necessarily require human intervention. For example, DAISY has been proposed for determining parameter identifiability of ODE models [48]; and the method of identifiability tableaus [49] is based on Jacobian matrix that involves partial derivatives, while our method is based on a system of polynomial equations and does not require the calculation of derivatives.

### Verification using benchmark models

In order to verify the validity of the proposed method, we have collected a number of benchmark models available in public literature to check whether the identifiability results obtained using our method are consistent with those obtained by other existing methods. Since these existing models do not contain any latent variable, we also consider a model with latent variables at the end of this section to show the capacity of our method.

The first benchmark model is for investigating the effects of smoking on lung cancer [24], the graph contains three nodes (variables), two directed edges, and one undirected edge (disturbance correlation). All the parameters in this model are found to be globally identifiable and the detailed analysis process have been shown in Fig. 6b. The second benchmark model was previously studied by Sullivant et al. [34], and its graph contains three nodes, one directed edge, and two undirected edges. Again, all the parameters in the second model turn out to be globally identifiable and the analysis details are given in Additional file 3. The third benchmark model investigated by Drton et al. [35] is for an acyclic graph with four nodes, three directed edges, and three undirected edges. From the same literature (Ref. [35]), we collected the fourth benchmark model that is more complicated in terms of number of variables and their interactions. The fifth benchmark model derived from the work of Kline el al. [22] is a cyclic graph with six nodes, six directed edges and three undirected edges. The purpose of this model is to show that the proposed approach can deal with cyclic graphs. We derived the sixth benchmark model from the work of Drton et al. [35]. This cyclic graph has six nodes, six directed edges, and three undirected edges; however, for this model, we also considered the case of multigraph (i.e., there exist both a directed edge and an undirected edge between two nodes), which has been paid particular attention in the previous study of Brito and Pearl [36]. We reported the structural identifiability analysis details and results of the third to sixth models also in Additional file 3.

**Fig. 7:**
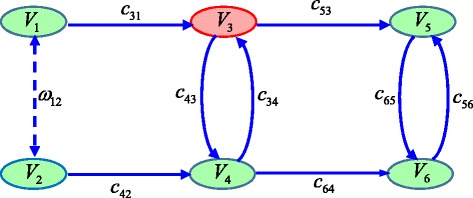
A mixed graph with feedback loops and one latent variable

While the identifiability results obtained using our method for all the benchmark models above are consistent with the conclusions in the existing literature, we have not found a model with explicit latent variables in literature. We thus derived such a model from the work of Kline [23] by assuming that node *V*_3_ is unobserved. As shown in Fig. 7, the mixed graph has 6 nodes, 2 cycles, and one latent variable *V*_3_ (labelled in red).

There are 9 parameters {*c*_31_, *c*_34_, *c*_42_, *c*_43_, *c*_53_, *c*_56_, *c*_64_, *c*_65_, *ω*_12_} in this model. Because the latent variable *Y*_3_ is not observed, the covariance between *Y*_3_ and other variables is unavailable for identifiability analysis. Therefore, only the following identifiability equations can be generated:

8 $$ \left\{\begin{array}{l}{\sigma}_{12}={\omega}_{12}\\ {}{\sigma}_{14}={c}_{31}{c}_{43}+{\omega}_{12}{c}_{42}\\ {}{\sigma}_{15}={c}_{31}{c}_{53}+{c}_{31}{c}_{43}{c}_{64}{c}_{56}+{\omega}_{12}{c}_{42}{c}_{34}{c}_{53}+{\omega}_{12}{c}_{42}{c}_{64}{c}_{56}\\ {}{\sigma}_{16}={c}_{31}{c}_{53}{c}_{65}+{c}_{31}{c}_{43}{c}_{64}+{\omega}_{12}{c}_{42}{c}_{64}+{\omega}_{12}{c}_{42}{c}_{34}{c}_{53}{c}_{65}\\ {}{\sigma}_{24}={c}_{42}+{\omega}_{12}{c}_{31}{c}_{43}\\ {}{\sigma}_{25}={c}_{42}{c}_{64}{c}_{56}+{c}_{42}{c}_{34}{c}_{53}+{\omega}_{12}{c}_{31}{c}_{53}+{\omega}_{12}{c}_{31}{c}_{43}{c}_{64}{c}_{56}\\ {}{\sigma}_{26}={c}_{42}{c}_{64}+{c}_{42}{c}_{34}{c}_{53}{c}_{65}+{\omega}_{12}{c}_{31}{c}_{53}{c}_{65}+{\omega}_{12}{c}_{31}{c}_{43}{c}_{64}\\ {}{\sigma}_{45}={c}_{34}{c}_{53}+{c}_{64}{c}_{56}\\ {}{\sigma}_{46}={c}_{64}+{c}_{34}{c}_{53}{c}_{65}\\ {}{\sigma}_{56}={c}_{56}\\ {}{\sigma}_{56}^{'}={c}_{65}\end{array}\right.. $$

**Fig. 8:**
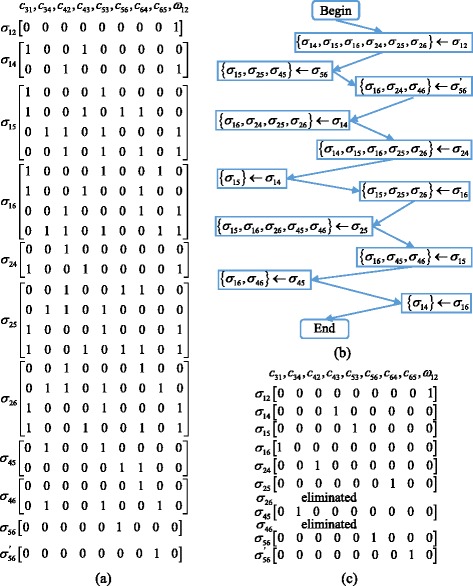
The reduction process of Example 2. The left arrow means that the left matrices are reduced by the right matrices. **a** The original identifiability matrices; **b** The reduction process; **c** The reduction results

The identifiability matrices in Fig. 8a can be generated according to the identifiability equations above, and these identifiability matrices are then reduced following the process shown in Fig. 8b. Finally, the reduction results in Fig. 8c are obtained, from which we can tell that the two matrices associated with *σ*_26_ and *σ*_46_ become empty and are labelled as eliminated. This observation suggests that there exist two redundant identifiability equations. Also, one can tell from Fig. 8c that all the other matrices have only one row with one “1” element. Therefore, all model parameters are globally identifiable despite the existence of a latent variable. This example model thus illustrates the capability of the proposed approach handling models with latent variables.

### Applications to real biological networks

Numerous biological networks can be found in a variety of databases or knowledge repositories [50, 51]; limited by resources, here we only consider a subnetwork structure of the within-host influenza virus life cycle as an application example. More specifically, influenza A virus (IAV) can infect multiple species including birds and human, and it has long been a major threat to public health by causing seasonal epidemics or sporadic pandemics [52]. A systematic understanding of IAV infection and immune response mechanisms is thus of significant scientific interest nowadays. For this purpose, a comprehensive map of the influenza virus life cycle together with molecular-level host responses has been previously constructed from hundreds of related publications by Matsuoka et al. [53], including several critical network modules like virus entry, virus replication and transcription, post-translational processing, transportation of virus proteins, and packaging and budding. Here we choose the subnetwork of virus replication, to which particular attention has been paid by many previous experimental studies [54–57].

**Fig. 9:**
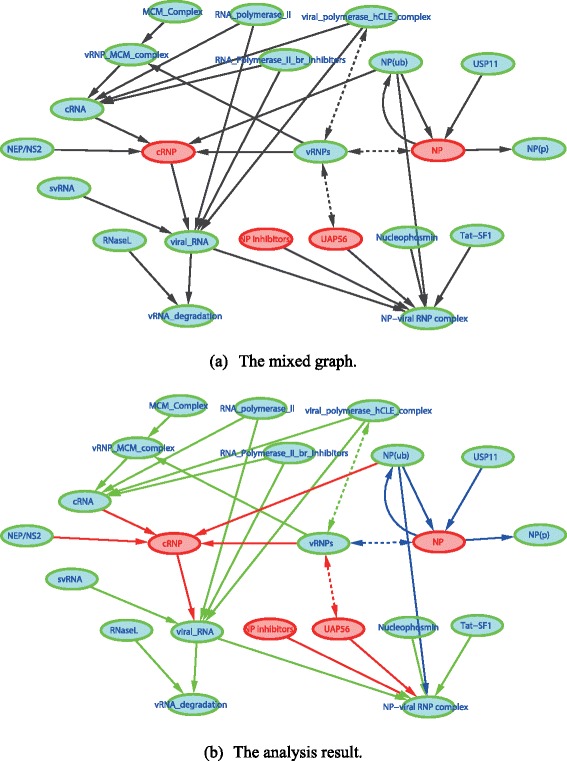
Identifiability analysis of the influenza A virus replication module. The read nodes are unobserved variables and the green nodes are observed variables in both **a** and **b**. In **b**, the globally identifiable edge coefficients are in green, the locally identifiable coefficients are in blue, and the unidentifiable coefficients are in red

However, influenza A virus replication is a complex process, involving many different biomolecules. It is therefore usually infeasible for one single experimental study to observe all the components and their interactions simultaneously, leading to the presence of latent variables. In addition, such complex molecular interactions cannot always be described by a directed acyclic graph due to the existence of, e.g., feedback loops. Therefore, we consider the IAV replication network module as a suitable example of cyclic graphical models with latent variables. We thus derived the mixed graph in Fig. 9a from Matsuoka’s work [53], which contains 22 nodes, 30 edges, and one cycle. The 5 pre-selected latent variables are labelled in red, and the observed nodes are in green. After applying the proposed algorithm to this network structure, the structure identifiability analysis result is visualized in Fig. 9b, where 16 globally identifiable edge coefficients are in green, 6 locally identifiable edge coefficients in blue, and 8 unidentifiable edge coefficients in red.

From the results in Fig. 9b, we can also tell that local network topological structures may have an important effect on parameter identifiability. For example, the NP inhibitor node has an in-degree 0 and is unobserved, which is the direct reason why all the edges starting from such a node are unidentifiable. In addition, both the cRNA and cRNP nodes have a comparatively high total degree (an in-degree 4 and an out-degree 1 for both nodes); however, the cRNP node is unobserved such that all the edges connected with it are unidentifiable, while the four incoming edges to the cRNA nodes are globally identifiable. The implication of such observations on experimental design is that, the nodes with an in-degree or out-degree 0 and the nodes with a high total degree (e.g., hub genes) are suggested to be experimentally observed to reduce the identifiability problem.

## Conclusions

In this study, we proposed a novel method for structural identifiability analysis of cyclic graphical models with explicit latent variables. Briefly, to deal with a broader range of network structures, the Wright’s path coefficient method is adapted to generate the identifiability equations and particular attention has been paid to cyclic mixed graphs (as well as the multigraph case, see Benchmark Model 5 in Additional file 3) with explicit latent variables. To achieve high computing efficiency, the identifiability equations are converted to binary identifiability matrices and the necessary strategies have been developed for matrix reduction and regrouping. Parameter identifiability can then be verified at the single parameter level based on the reduced and grouped identifiability matrices after a connection between the number of non-zero matrix elements and the theoretical work of Garcia and Li. The validity of the proposed approach was theoretically justified and further verified using existing benchmark models. In addition, the proposed approach was applied to a real network structure for influenza A virus replication to gain insights into experimental design.

In summary, this study provides a basis for efficient model refinement and informative experiment design, and thus may facilitate investigators to expedite our understanding of network structure and interaction mechanisms in complex biological systems. However, we recognize that many real biological networks are high-dimensional with complex nonlinear interactions. Therefore, the proposed approach will need to be extended to deal with more realistic problems in the future.

## Abbreviations

DAG, directed acyclic graph; IAV, influenza A virus; ODE, ordinary differential equation; SEM, structure equation model

## Additional files

Additional file 1: Identifiability Preservation by Matrix Reduction. This file contains the theoretical justification for the proposed identifiability matrix reduction operations. (PDF 92 kb) Additional file 2: Proof of Theorem 1. This file includes the details of theoretical derivation of Theorem 1. (PDF 62 kb) Additional file 3: Validation Using Benchmark Models. This file contains 5 benchmark models selected from related literature for verifying the validity of the proposed method. (PDF 100 kb)
