# Supplementary material for: Structural identifiability of cyclic graphical models of biological networks with latent variables
Source: BMC Syst Biol. 2016 Jun 13;10:41. doi: 10.1186/s12918-016-0287-y (PMC4906697; doi:10.1186/s12918-016-0287-y)
Supplement: Additional file 1: — Identifiability Preservation by Matrix Reduction. This file contains the theoretical justification for the proposed identifiability matrix reduction operations. (PDF 92 kb) [file 12918_2016_287_MOESM1_ESM.pdf]

Supporting Materials I: Identifiability Preservation by Matrix Reduction

In this study, the identifiability equations of a SEM are generated by Wright's path coefficient method. That is, the covariance σ_{ij} between a pair of variables V_i and V_j is equal to $\sum_{path_k}\prod_{edge_l}\theta_l$. Note that each monomial $\prod_{edge_l}\theta_l$ corresponds to an non-redundant path between V_i and V_j, thus there exist no identical monomials $\prod_{edge_l}\theta_l$ in all the identifiability equations. This observation also suggests that none of the identifiability equations can be expressed in terms of linear combinations of other equations, which is called the non-redundancy property.

Before we show the identifiability preservation by identifiability matrix reduction, the following definitions need to be introduced.

Definition 1 (Equivalent Identifiability Equations) If two identifiability equations $f_1(\theta)$ and $f_2(\theta)$ can be reduced to the same Gröbner basis, then $f_1(\theta)$ and $f_2(\theta)$ are called equivalent, denoted by $f_1(\theta) \sim f_2(\theta)$. □

Definition 2 (Equivalent Identifiability Matrices) For two identifiability matrices $\mathbf{M}_1$ and $\mathbf{M}_2$, if the two corresponding identifiability equations are equivalent, then $\mathbf{M}_1$ and $\mathbf{M}_2$ are also equivalent, denoted by $\mathbf{M}_1 \sim \mathbf{M}_2$. □

Remark 1. According to Definition 1, it is straightforward to tell that addition or multiplication of a constant to a monomial term will produce an equivalent equation. For example, given $f_1(\omega,c)$: $\sigma_{12} = a_1\omega_{12} + a_2c_{31}\omega_{23}$, $f_2(\omega,c)$: $\sigma_{12} - 5 = a_1\omega_{12} + a_2c_{31}\omega_{23}$, $f_3(\omega,c)$: $\sigma_{12} = 3a_1\omega_{12} + a_2c_{31}\omega_{23}$ and $f_4(\omega,c)$: $\sigma_{12} - 2 = 3a_1\omega_{12} + 4a_2c_{31}\omega_{23}$, where a_1 and a_2 are the constant coefficients of

monomials, then $f_1(\omega,c) \sim f_2(\omega,c) \sim f_3(\omega,c) \sim f_4(\omega,c)$. □

According to Definition 2, we can introduce three categories of operations on identifiability matrices, which will preserve the identifiability of the original system.

i) **Row swap**. Let $\boldsymbol{R}_i$ and $\boldsymbol{R}_j$ $(i \neq j)$ denote two different rows of an identifiability matrix $\mathbf{M}_1$, and let $\mathbf{M}_2$ denote the matrix generated after swapping $\boldsymbol{R}_i$ and $\boldsymbol{R}_j$, then $\mathbf{M}_1 \sim \mathbf{M}_2$.

Proof. According to the generation rule of identifiability matrices, the row $\boldsymbol{R}_i$ represents the *i*-th monomial $\prod_{edge_l} \theta_l^{(i)}$ of an identifiability equation $\sigma = \sum_{path_k} \prod_{edge_l} \theta_l$; similarly, the row $\boldsymbol{R}_j$ represents the *j*-th monomial $\prod_{edge_l} \theta_l^{(j)}$ of the same identifiability equation. Swapping two rows $\boldsymbol{R}_i \leftrightarrow \boldsymbol{R}_j$ is equivalent to swap the positions of the two monomials in the identifiability equation, which will not change the identifiability equation according to the communitive law $\prod_{edge_l} \theta_l^{(i)} + \prod_{edge_l} \theta_l^{(j)} = \prod_{edge_l} \theta_l^{(j)} + \prod_{edge_l} \theta_l^{(i)}$. Therefore, $\mathbf{M}_1$ is equivalent to $\mathbf{M}_2$. ■

ii) **Redundant row removal**. Let $\boldsymbol{R}_i$ and $\boldsymbol{R}_j$ $(i \neq j)$ denote two different rows of an identifiability matrix $\mathbf{M}_1$. If $\boldsymbol{R}_i = \boldsymbol{R}_j$ and let $\mathbf{M}_2$ denote the matrix generated after removing $\boldsymbol{R}_i$ or $\boldsymbol{R}_j$, then $\mathbf{M}_1 \sim \mathbf{M}_2$.

Proof. If $\boldsymbol{R}_i = \boldsymbol{R}_j$, the corresponding monomials are the same (maybe expect for the constant coefficients in front). The two monomials can thus be merged into one monomial term, which indicates that $\mathbf{M}_2$ is equivalent to $\mathbf{M}_1$. ■

iii) **Row deletion**. Let $\mathbf{M}_1$ and $\mathbf{M}_2$ be two identifiability matrices, which

correspond to two different identifiability equations, such that $N_R\left(\mathbf{M}_1\right)>1$ and $\mathbf{M}_2 \subseteq \mathbf{M}_1$. Also, let $\mathbf{M}_3 = sub(\mathbf{M}_1)$ be a submatrix consisting of $\mathbf{M}_1$'s rows that $\mathbf{M}_2$ has in $\mathbf{M}_1$. See Fig. 3 for examples.

- If $Rem\left(\mathbf{M}_{1-2}\right) \neq \mathbf{M}_Z$ and $Comp\left(\mathbf{M}_3 - \mathbf{M}_2\right) = \mathbf{M}_Z$, then $\mathbf{M}_1$ can be reduced to $Rem(\mathbf{M}_{1-2})$ without altering the parameter identifiability;
- If $Rem(\mathbf{M}_{1-2}) \neq \mathbf{M}_Z$ and $Comp\left(\mathbf{M}_3 - \mathbf{M}_2\right) = \mathbf{M}_R$, then $\mathbf{M}_1$ can be reduced to $\begin{bmatrix} Rem(\mathbf{M}_{1-2}) \\ M_{RI} \end{bmatrix}$ without altering the parameter identifiability;
- If $Rem\left(\mathbf{M}_{1-2}\right) = \mathbf{M}_Z$ and $Comp\left(\mathbf{M}_3 - \mathbf{M}_2\right) = \mathbf{M}_R$, then $\mathbf{M}_1$ can be reduced to $\mathbf{M}_{RI}$ without altering the parameter identifiability;
- If $Rem\left(\mathbf{M}_{1-2}\right) = \mathbf{M}_Z$ and $Comp\left(\mathbf{M}_3 - \mathbf{M}_2\right) = \mathbf{M}_z$ (i.e. $\mathbf{M}_1 = \mathbf{M}_2 = \mathbf{M}_3$), and take the row which has the least "1" elements in $\mathbf{M}_1$ to form a new matrix $\mathbf{M}_4$, then $\mathbf{M}_1$ can be reduced to $\mathbf{M}_4$ without altering the parameter identifiability.

Proof. (1) If $Rem\left(\mathbf{M}_{1-2}\right) \neq \mathbf{M}_Z$ and $Comp\left(\mathbf{M}_3 - \mathbf{M}_2\right) = \mathbf{M}_Z$, we know that $\mathbf{M}_1$ consists of $\mathbf{M}_2$ and $Rem(\mathbf{M}_{1-2})$. That is, the identifiability equation corresponding to $\mathbf{M}_1$ can be divided into two parts. The part corresponding to $\mathbf{M}_2$ can be denoted as $expr\left(\mathbf{M}_2\right)$, and the other part corresponding to $Rem(\mathbf{M}_{1-2})$ can be denoted as $expr\left(Rem(\mathbf{M}_{1-2})\right)$. Let σ_1 and σ_2 denote the covariance corresponding to $\mathbf{M}_1$ and $\mathbf{M}_2$, respectively, then $\sigma_1 = expr\left(\mathbf{M}_2\right) + expr\left(Rem(\mathbf{M}_{1-2})\right)$ and $\sigma_2 = expr\left(\mathbf{M}_2\right)$. Using simple algebraic operations, we obtain $\sigma_1 - \sigma_2 = expr\left(Rem(\mathbf{M}_{1-2})\right)$. Since σ_2 is a known constant, according to Remark 1, we know that $\mathbf{M}_1$ can be reduced to $Rem(\mathbf{M}_{1-2})$ without altering the parameter identifiability.

(2) Let σ_1 and σ_2 be the known covariance corresponding to $\mathbf{M}_1$ and $\mathbf{M}_2$, respectively, that is, $\sigma_1 = expr(M_1)$ and $\sigma_2 = expr(M_2)$. If $Rem(\mathbf{M}_{1-2}) \neq \mathbf{M}_Z$ and $Comp\left(\mathbf{M}_3 - \mathbf{M}_2\right) = \mathbf{M}_R$, then certain monomials in the identifiability equation corresponding to $\mathbf{M}_1$ will have a common term such that $\sigma_1 = expr\left(Rem(\mathbf{M}_{1-2})\right) + expr\left(\mathbf{M}_{RI}\right) \times expr\left(\mathbf{M}_2\right)$. Replace $expr\left(\mathbf{M}_2\right)$ with σ_2 and obtain $\sigma_1 = expr\left(Rem(\mathbf{M}_{1-2})\right) + \sigma_2 expr\left(\mathbf{M}_{RI}\right)$. It is known from Lemma 1 that changing the coefficient of a monomial does not change its identifiability matrix of an identifiability equation. So $\mathbf{M}_1$ can be reduced to $\begin{bmatrix} Rem(\mathbf{M}_{1-2}) \\ M_{RI} \end{bmatrix}$ without altering the parameter identifiability.

(3) Let σ_1 and σ_2 be the known covariance corresponding to $\mathbf{M}_1$ and $\mathbf{M}_2$, respectively, that is, $\sigma_1 = expr\left(\mathbf{M}_1\right)$ and $\sigma_2 = expr\left(\mathbf{M}_2\right)$. If $Rem(\mathbf{M}_{1-2}) = \mathbf{M}_Z$ and $Comp\left(\mathbf{M}_3 - \mathbf{M}_2\right) = \mathbf{M}_R$, then all the monomials in the identifiability equation corresponding to $\mathbf{M}_1$ share a common term such that $\sigma_1 = expr\left(\mathbf{M}_{RI}\right) \times expr\left(\mathbf{M}_2\right)$. Replace $expr\left(\mathbf{M}_2\right)$ by σ_2 to obtain $\sigma_1 = \sigma_2 expr\left(\mathbf{M}_{RI}\right)$. According to Lemma 1, changing the coefficient of a monomial does not change its identifiability matrix in. Therefore, $\mathbf{M}_1$ can be reduced to $\mathbf{M}_{RI}$ without altering the parameter identifiability.

(4) If $Rem(\mathbf{M}_{1-2}) = \mathbf{M}_Z$ and $Comp\left(\mathbf{M}_3 - \mathbf{M}_2\right) = \mathbf{M}_z$, we have $\mathbf{M}_1 = \mathbf{M}_2 = \mathbf{M}_3$. The matrix $\mathbf{M}_4$ has only one row, which is the row with the least number of ones in $\mathbf{M}_1$, so we can express $\mathbf{M}_1$ as $\mathbf{M}_1 = \begin{bmatrix} \mathbf{M}_4 \\ Rem(\mathbf{M}_{1-4}) \end{bmatrix}$. Now let σ_1 and σ_2 be the known covariance corresponding to $\mathbf{M}_1$ and $\mathbf{M}_2$, respectively, then we have

$$\sigma_1 = a_1 \cdot expr\left(\mathbf{M}_4\right) + a_2 \cdot expr\left(Rem(\mathbf{M}_{1-4})\right),$$

$$\sigma_2 = b_1 \cdot expr\left(\mathbf{M}_4\right) + b_2 \cdot expr\left(Rem(\mathbf{M}_{1-4})\right),$$

where a_1, a_2, b_1 and b_2 are the nonzero constant coefficients. It is known from the non-redundant property that these two equations are linearly independent. So from the latter equation we have $expr\left(Rem(\mathbf{M}_{1-4})\right) = \sigma_2 / b_2 - b_1 / b_2 \times expr\left(\mathbf{M}_4\right)$. Replace $expr\left(Rem\left(\mathbf{M}_{1\text{-}4}\right)\right)$ by $\sigma_2 / b_2 - b_1 / b_2 \times expr\left(\mathbf{M}_4\right)$ in the equation for σ_1 to obtain

$$\sigma_1 = a_1 \times expr\left(\mathbf{M}_4\right) + a_2\sigma_2 / b_2 - a_2 b_1 / b_2 \times expr\left(\mathbf{M}_4\right). \tag{I.1}$$

Thus, we can rewrite this equation as

$$\sigma_3 = a_3 \times expr\left(\mathbf{M}_4\right), \tag{I.2}$$

where $\sigma_3 = \sigma_1 - a_2\sigma_2 / b_1$, $a_3 = a_1 - a_2 b_1 / b_2$ and $a_3 \neq 0$. Since (I.1) and (I.2) have the same Gröbner basis, we know $\mathbf{M}_1$ can be reduced to $\mathbf{M}_4$ without altering the parameter identifiability. ■
