# Supplementary material for: Structural identifiability of cyclic graphical models of biological networks with latent variables
Source: BMC Syst Biol. 2016 Jun 13;10:41. doi: 10.1186/s12918-016-0287-y (PMC4906697; doi:10.1186/s12918-016-0287-y)
Supplement: Additional file 2: — Proof of Theorem 1. This file includes the details of theoretical derivation of Theorem 1. (PDF 62 kb) [file 12918_2016_287_MOESM2_ESM.pdf]

Supporting Materials II —— Proof of Theorem 1

Theorem 1 For the reduced identifiability matrices in the same group, let N_M denote the number of matrices, let N_P denote the number of unknown parameters, and let $N_{\max}$ be the maximum number of the "1" elements in one row of all the matrices.

- When $N_P > N_M$, all the parameters in the same group are unidentifiable;
- When $N_P = N_M$, the parameters are globally identifiable if $N_{\max} = 1$, and locally identifiable if $N_{\max} > 1$;
- When $N_P < N_M$, the parameters are at least locally identifiable.

Proof. By definition of structural identifiability, the number of solutions to the identifiability equations is the key to verify parameter identifiability. According to the generation rule of identifiability matrices, N_M identifiability matrices correspond to N_M different identifiability equations. If the number of matrices is less than the number of unknown parameters (that is, $N_M < N_P$), the number of polynomial symbolic equations is then less than the number of unknown parameters. Because the identifiability equations are in the form of $\sigma_{ij} = \sum_{path_k} \prod_{edge_l} \theta_l$ and the order of θ_l is at most one in each monomial, this leads to an underdetermined system such that there exist an infinite number of solutions. Therefore, all the parameters are unidentifiable if the number of matrices is less than the number of parameters.

If the number of matrices is equal to the number of parameters in a group (i.e., $N_M = N_P$), then we have N_P polynomial equations for real/complex variables with

N_P unknown parameters. According to the work of Garcia and Li [1], the number of solutions is equal to $q = \prod_{i=1}^{N_p} q_i$, where q_i is the degree (the power of the highest order term) of equation i. If the maximum number $N_{\max}$ of the "1" elements in one row of all the matrices is equal to 1, then $q = 1$; this means that each parameter has a unique solution. In other words, every parameter is globally identifiable in this group. If the number $N_{\max}$ is greater than 1, then $q > 1$; this means there exist a finite number of solutions. In other words, every parameter is locally identifiable in the same group.

If the number of matrices is greater than the number of parameters in a group (i.e., $N_P < N_M$), then the equations corresponding to the matrices form an overdetermined polynomial system. For such a system, the number of solutions cannot be determined because even if we only change the constants in the system (e.g., constant coefficients), the number of solutions can be zero, one or a finite number, which needs to be analyzed case by case. However, from the parameter estimation point of view (i.e., the original problem becomes an optimization problem), there exist multiple local solutions [2-5]. Therefore, when $N_P < N_M$, the parameters are at least locally identifiable. ■

References

1. Garcia C, Li T. On the Number of Solutions to Polynomial Systems of Equations. SIAM Journal on Numerical Analysis. 1979.
2. Dedieu J, Shub M. Newton's method for overdetermined systems of equations.

Mathematics of Computation of the American Mathematical Society. 2000;69(231):1099-115.
3. Bardet M, Faugere J-C, Salvy B, editors. On the complexity of Gröbner basis computation of semi-regular overdetermined algebraic equations. Proceedings of the International Conference on Polynomial System Solving; 2004.
4. Lichtblau D. Approximate Gröbner Bases, Overdetermined Polynomial Systems, and Approximate GCDs. ISRN Computational Mathematics. 2013.
5. Amoroso F, Leroux L, Sombra M. Overdetermined systems of sparse polynomial equations. Foundations of Computational Mathematics. 2015;15(1):53-87.
