# Supplementary material for: Structural identifiability of cyclic graphical models of biological networks with latent variables
Source: BMC Syst Biol. 2016 Jun 13;10:41. doi: 10.1186/s12918-016-0287-y (PMC4906697; doi:10.1186/s12918-016-0287-y)
Supplement: Additional file 3: — Validation Using Benchmark Models. This file contains 5 benchmark models selected from related literature for verifying the validity of the proposed method. (PDF 100 kb) [file 12918_2016_287_MOESM3_ESM.pdf]

Supporting Materials III —— Validation Using Benchmark Models

Benchmark Model 1. See Fig. 6(b) in the manuscript.

Benchmark Model 2. Consider the mixed graph in Fig. S-1, which has been studied by Sullivant [1].

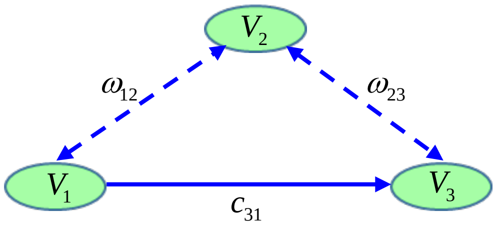

Figure S-1. A mixed graph with three nodes.

There are three parameters in this model, and the identifiability equations are as follows

$$\begin{cases} \sigma_{12} = \omega_{12} + c_{31}\omega_{23} \\ \sigma_{13} = c_{31} \\ \sigma_{23} = \omega_{23} + \omega_{12}c_{31} \end{cases}.$$

Let the column names of the identifiability matrices be $\{c_{31}, \omega_{12}, \omega_{23}\}$, and the identifiability matrices are generated from the identifiability equations as follows

$$\sigma_{12}\begin{bmatrix} 0 & 1 & 0 \\ 1 & 0 & 1 \end{bmatrix},$$

$$\sigma_{13}\begin{bmatrix} 1 & 0 & 0 \end{bmatrix},$$

$$\sigma_{23}\begin{bmatrix} 0 & 0 & 1 \\ 1 & 1 & 0 \end{bmatrix}.$$

Reduce matrices σ_{12} and σ_{23} by matrix σ_{13} separately and get matrices

$$\sigma_{12}\begin{bmatrix} 0 & 1 & 0 \\ 0 & 0 & 1 \end{bmatrix},$$

$\sigma_{23}\begin{bmatrix} 0 & 0 & 1 \\ 0 & 1 & 0 \end{bmatrix}$.

Use the row deletion operation to reduce matrix σ_{12} by matrix σ_{23} and obtain the simplified matrix $\sigma_{12}\begin{bmatrix} 0 & 0 & 1 \end{bmatrix}$. Then reduce matrix σ_{23} by matrix σ_{12} and get $\sigma_{23}\begin{bmatrix} 0 & 1 & 0 \end{bmatrix}$. Now the reduction process is finished, and each matrix has only one row with only one "1" element. Therefore, all parameters are globally identifiable in this model.

Benchmark Model 3. Consider the mixed graph in Fig. S-2, which has been studied by Drton [2].

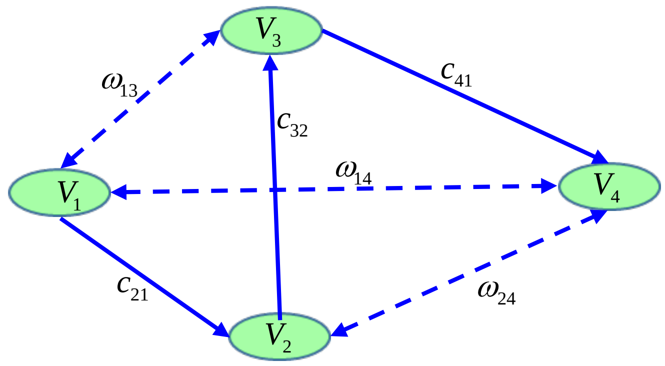

Figure S-2. A mixed graph with four nodes.

There are six parameters in this model and its identifiability equations are

$$\begin{cases} \sigma_{12} = c_{21} \\ \sigma_{13} = \omega_{13} + c_{21}c_{32} \\ \sigma_{14} = \omega_{14} + \omega_{13}c_{43} + c_{21}c_{32}c_{43} + c_{21}\omega_{24} \\ \sigma_{23} = c_{32} \\ \sigma_{24} = c_{32}c_{43} + \omega_{24} \\ \sigma_{34} = c_{43} \end{cases}.$$

The columns of the identifiability matrices correspond to $\{c_{21}, c_{32}, c_{43}, \omega_{13}, \omega_{14}, \omega_{24}\}$, respectively, and the identifiability matrices become

$$\sigma_{12}\begin{bmatrix}1 & 0 & 0 & 0 & 0 & 0\end{bmatrix},$$

$$\sigma_{13}\begin{bmatrix}0 & 0 & 0 & 1 & 0 & 0\\ 1 & 1 & 0 & 0 & 0 & 0\end{bmatrix},$$

$$\sigma_{14}\begin{bmatrix}0 & 0 & 0 & 0 & 1 & 0\\ 0 & 0 & 1 & 1 & 0 & 0\\ 1 & 1 & 1 & 0 & 0 & 0\\ 1 & 0 & 0 & 0 & 0 & 1\end{bmatrix},$$

$$\sigma_{23}\begin{bmatrix}0 & 1 & 0 & 0 & 0 & 0\end{bmatrix},$$

$$\sigma_{24}\begin{bmatrix}0 & 1 & 1 & 0 & 0 & 0\\ 0 & 0 & 0 & 0 & 0 & 1\end{bmatrix},$$

$$\sigma_{34}\begin{bmatrix}0 & 0 & 1 & 0 & 0 & 0\end{bmatrix}.$$

Reduce matrices σ_{13}, σ_{14} and σ_{24} by matrices σ_{12}, σ_{23} and σ_{34}, respectively, and we obtain

$$\sigma_{13}\begin{bmatrix}0 & 0 & 0 & 1 & 0 & 0\end{bmatrix},$$

$$\sigma_{14}\begin{bmatrix}0 & 0 & 0 & 0 & 1 & 0\\ 0 & 0 & 0 & 1 & 0 & 0\\ 0 & 0 & 0 & 0 & 0 & 1\end{bmatrix},$$

$$\sigma_{24}\begin{bmatrix}0 & 0 & 0 & 0 & 0 & 1\end{bmatrix}.$$

Further reduce matrix σ_{14} by σ_{13} and σ_{24} to obtain $\sigma_{14}\begin{bmatrix}0 & 0 & 0 & 0 & 1 & 0\end{bmatrix}$. Now the reduction process is finished, and each matrix has only one row with only one "1" element. Therefore, this model is globally identifiable.

Benchmark Model 4. Consider the mixed graph in Fig. S-3, which has also been studied by Drton [2].

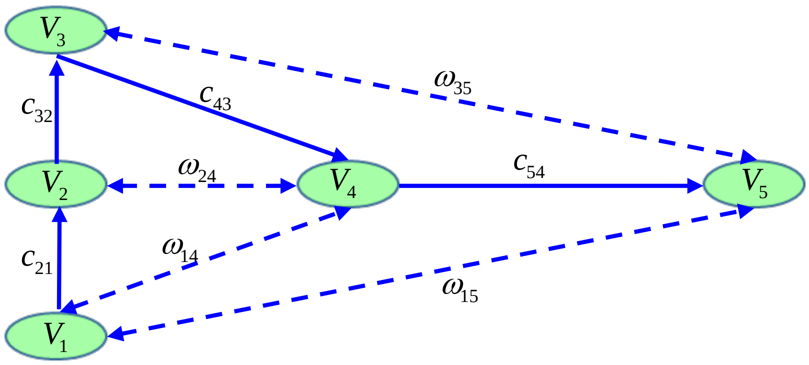

Figure S-3. A mixed graph with five nodes.

There are eight parameters in this model and the following identifiability equations can be generated

$$\begin{cases} \sigma_{12} = c_{21} \\ \sigma_{13} = c_{21}c_{32} \\ \sigma_{14} = \omega_{14} + c_{21}c_{32}c_{43} + c_{21}\omega_{24} \\ \sigma_{15} = \omega_{15} + \omega_{14}c_{54} + c_{21}\omega_{24}c_{54} + c_{21}c_{32}\omega_{35} + c_{21}c_{32}c_{43}c_{54} \\ \sigma_{23} = c_{32} \\ \sigma_{24} = c_{32}c_{43} + \omega_{24} \\ \sigma_{25} = \omega_{24}c_{54} + c_{32}\omega_{35} + c_{32}c_{43}c_{54} \\ \sigma_{34} = c_{43} \\ \sigma_{35} = \omega_{35} + c_{43}c_{54} \\ \sigma_{45} = c_{54} \end{cases}.$$

The columns of the identifiability matrices correspond to $\{c_{21}, c_{32}, c_{43}, c_{54}, \omega_{14}, \omega_{15}, \omega_{24}, \omega_{35}\}$, respectively, and the identifiability matrices can be derived as follows

$$\sigma_{12}\begin{bmatrix}1 & 0 & 0 & 0 & 0 & 0 & 0 & 0\end{bmatrix},$$

$$\sigma_{13}\begin{bmatrix}1 & 1 & 0 & 0 & 0 & 0 & 0 & 0\end{bmatrix},$$

$$\sigma_{14}\begin{bmatrix}0 & 0 & 0 & 0 & 1 & 0 & 0 & 0 \\ 1 & 1 & 1 & 0 & 0 & 0 & 0 & 0 \\ 1 & 0 & 0 & 0 & 0 & 0 & 1 & 0\end{bmatrix},$$

$$\sigma_{15}\begin{bmatrix}0&0&0&0&0&1&0&0\\0&0&0&1&1&0&0&0\\1&0&0&1&0&0&1&0\\1&1&0&0&0&0&0&1\\1&1&1&1&0&0&0&0\end{bmatrix},$$

$$\sigma_{23}\begin{bmatrix}0&1&0&0&0&0&0&0\end{bmatrix},$$

$$\sigma_{24}\begin{bmatrix}0&1&1&0&0&0&0&0\\0&0&0&0&0&0&1&0\end{bmatrix},$$

$$\sigma_{25}\begin{bmatrix}0&0&0&1&0&0&1&0\\0&1&0&0&0&0&0&1\\0&1&1&1&0&0&0&0\end{bmatrix},$$

$$\sigma_{34}\begin{bmatrix}0&0&1&0&0&0&0&0\end{bmatrix},$$

$$\sigma_{35}\begin{bmatrix}0&0&0&0&0&0&0&1\\0&0&1&1&0&0&0&0\end{bmatrix},$$

$$\sigma_{45}\begin{bmatrix}0&0&0&1&0&0&0&0\end{bmatrix}.$$

Reduce matrix σ_{13} by matrix σ_{12} to obtain the simplest form as follows

$$\sigma_{13}\begin{bmatrix}0&1&0&0&0&0&0&0\end{bmatrix}.$$

Then reduce matrices σ_{14}, σ_{15}, σ_{24}, σ_{25} and σ_{35} by matrices σ_{12}, σ_{13}, σ_{23} and σ_{45}, respectively, to obtain

$$\sigma_{14}\begin{bmatrix}0&0&0&0&1&0&0&0\\0&0&0&0&0&0&1&0\end{bmatrix},$$

$$\sigma_{15}\begin{bmatrix}0&0&0&0&0&1&0&0\\0&0&0&0&1&0&0&0\\0&0&0&0&0&0&1&0\\0&0&0&0&0&0&0&1\end{bmatrix},$$

$$\sigma_{24}\begin{bmatrix}0&0&0&0&0&0&1&0\end{bmatrix},$$

$$\sigma_{25}\begin{bmatrix}0&0&0&0&0&0&1&0\\0&0&0&0&0&0&0&1\end{bmatrix},$$

$$\sigma_{35}\begin{bmatrix}0&0&0&0&0&0&0&1\end{bmatrix}.$$

Now reduce matrices σ_{14}, σ_{15} and σ_{25} by matrices σ_{24} and σ_{35}, and we get

$\sigma_{14}\begin{bmatrix}0 & 0 & 0 & 0 & 1 & 0 & 0 & 0\end{bmatrix}$,

$$\sigma_{15}\begin{bmatrix}0 & 0 & 0 & 0 & 0 & 1 & 0 & 0\\ 0 & 0 & 0 & 0 & 1 & 0 & 0 & 0\end{bmatrix}.$$

Note that matrix σ_{25} becomes empty and thus dropped from further analysis. Then reduce matrix σ_{15} by matrix σ_{14} to obtain $\sigma_{15}\begin{bmatrix}0 & 0 & 0 & 0 & 0 & 1 & 0 & 0\end{bmatrix}$. Now each matrix has only one row that has only one "1" element. Therefore, this model is globally identifiable.

Benchmark Model 5. Consider the mixed graph in Fig. S-4, which has been studied by Kline [3]. Note that the measurement model of the original graph is ignored here because it is not the focus of this study.

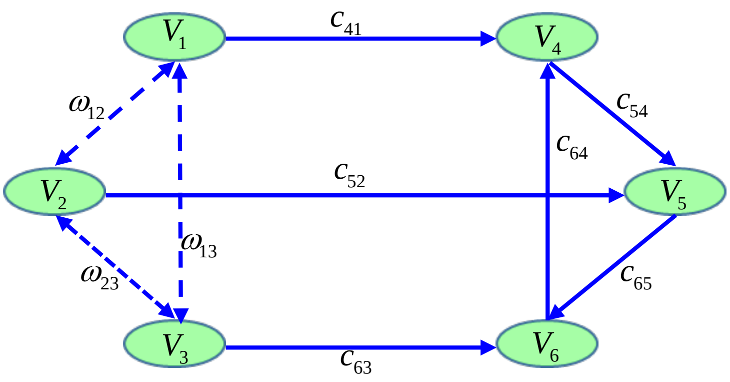

Figure S-4. A mixed graph with six nodes and circles.

There are nine parameters in this model and its identifiability equations are as follows

$$\begin{cases} \sigma_{12} = \omega_{12} \\ \sigma_{13} = \omega_{13} \\ \sigma_{14} = c_{41} + \omega_{12}c_{52}c_{65}c_{46} + \omega_{13}c_{63}c_{46} \\ \sigma_{15} = c_{41}c_{54} + \omega_{12}c_{52} + \omega_{13}c_{63}c_{46}c_{54} \\ \sigma_{16} = c_{41}c_{54}c_{65} + \omega_{12}c_{52}c_{65} + \omega_{13}c_{63} \\ \sigma_{23} = \omega_{23} \\ \sigma_{24} = \omega_{12}c_{41} + c_{52}c_{65}c_{46} + \omega_{23}c_{63}c_{46} \\ \sigma_{25} = c_{52} + \omega_{12}c_{41}c_{54} + \omega_{23}c_{63}c_{46}c_{54} \\ \sigma_{26} = c_{52}c_{65} + \omega_{23}c_{63} + \omega_{12}c_{41}c_{54}c_{65} \\ \sigma_{34} = c_{63}c_{46} + \omega_{23}c_{52}c_{65}c_{46} + \omega_{13}c_{41} \\ \sigma_{35} = c_{63}c_{46}c_{54} + \omega_{13}c_{41}c_{54} + \omega_{23}c_{52} \\ \sigma_{36} = c_{63} + \omega_{23}c_{52}c_{65} + \omega_{13}c_{41}c_{54}c_{65} \\ \sigma_{45} = c_{54} \\ \sigma_{45} = c_{65}c_{46} \\ \sigma_{46} = c_{54}c_{65} \\ \sigma_{46} = c_{46} \\ \sigma_{56} = c_{65} \\ \sigma_{56} = c_{46}c_{54} \end{cases}.$$

The columns of the identifiability matrices correspond to $\{c_{41}, c_{52}, c_{63}, c_{46}, c_{54}, c_{65}, \omega_{12}, \omega_{13}, \omega_{23}\}$, respectively, and the identifiability matrices can be generated as follows

$$\sigma_{12}\begin{bmatrix}0 & 0 & 0 & 0 & 0 & 0 & 1 & 0 & 0\end{bmatrix},$$

$$\sigma_{13}\begin{bmatrix}0 & 0 & 0 & 0 & 0 & 0 & 0 & 1 & 0\end{bmatrix},$$

$$\sigma_{14}\begin{bmatrix}1 & 0 & 0 & 0 & 0 & 0 & 0 & 0 & 0 \\ 0 & 1 & 0 & 1 & 0 & 1 & 1 & 0 & 0 \\ 0 & 0 & 1 & 1 & 0 & 0 & 0 & 1 & 0\end{bmatrix},$$

$$\sigma_{15}\begin{bmatrix}1 & 0 & 0 & 0 & 1 & 0 & 0 & 0 & 0 \\ 0 & 1 & 0 & 0 & 0 & 0 & 1 & 0 & 0 \\ 0 & 0 & 1 & 1 & 1 & 0 & 0 & 1 & 0\end{bmatrix},$$

$$\sigma_{16}\begin{bmatrix}1 & 0 & 0 & 0 & 1 & 1 & 0 & 0 & 0 \\ 0 & 1 & 0 & 0 & 0 & 1 & 1 & 0 & 0 \\ 0 & 0 & 1 & 0 & 0 & 0 & 0 & 1 & 0\end{bmatrix},$$

$$\sigma_{23}\begin{bmatrix}0 & 0 & 0 & 0 & 0 & 0 & 0 & 0 & 1\end{bmatrix},$$

$$\sigma_{24}\begin{bmatrix}1 & 0 & 0 & 0 & 0 & 0 & 1 & 0 & 0\\ 0 & 1 & 0 & 1 & 0 & 1 & 0 & 0 & 0\\ 0 & 0 & 1 & 1 & 0 & 0 & 0 & 0 & 1\end{bmatrix},$$

$$\sigma_{25}\begin{bmatrix}0 & 1 & 0 & 0 & 0 & 0 & 0 & 0 & 0\\ 1 & 0 & 0 & 0 & 1 & 0 & 1 & 0 & 0\\ 0 & 0 & 1 & 1 & 1 & 0 & 0 & 0 & 1\end{bmatrix},$$

$$\sigma_{26}\begin{bmatrix}0 & 1 & 0 & 0 & 0 & 1 & 0 & 0 & 0\\ 0 & 0 & 1 & 0 & 0 & 0 & 0 & 0 & 1\\ 1 & 0 & 0 & 0 & 1 & 1 & 1 & 0 & 0\end{bmatrix},$$

$$\sigma_{34}\begin{bmatrix}0 & 0 & 1 & 1 & 0 & 0 & 0 & 0 & 0\\ 0 & 1 & 0 & 1 & 0 & 1 & 0 & 0 & 1\\ 1 & 0 & 0 & 0 & 0 & 0 & 0 & 1 & 0\end{bmatrix},$$

$$\sigma_{35}\begin{bmatrix}0 & 0 & 1 & 1 & 1 & 0 & 0 & 0 & 0\\ 1 & 0 & 0 & 0 & 1 & 0 & 0 & 1 & 0\\ 0 & 1 & 0 & 0 & 0 & 0 & 0 & 0 & 1\end{bmatrix},$$

$$\sigma_{36}\begin{bmatrix}0 & 0 & 1 & 0 & 0 & 0 & 0 & 0 & 0\\ 0 & 1 & 0 & 0 & 0 & 1 & 0 & 0 & 1\\ 1 & 0 & 0 & 0 & 1 & 1 & 0 & 1 & 0\end{bmatrix},$$

$$\sigma_{45}\begin{bmatrix}0 & 0 & 0 & 0 & 1 & 0 & 0 & 0 & 0\end{bmatrix},$$

$$\sigma_{45}\begin{bmatrix}0 & 0 & 0 & 1 & 0 & 1 & 0 & 0 & 0\end{bmatrix},$$

$$\sigma_{46}\begin{bmatrix}0 & 0 & 0 & 1 & 1 & 0 & 0 & 0 & 0\end{bmatrix},$$

$$\sigma_{46}\begin{bmatrix}0 & 0 & 0 & 1 & 0 & 0 & 0 & 0 & 0\end{bmatrix},$$

$$\sigma_{56}\begin{bmatrix}0 & 0 & 0 & 0 & 0 & 1 & 0 & 0 & 0\end{bmatrix},$$

$$\sigma_{56}\begin{bmatrix}0 & 0 & 0 & 1 & 1 & 0 & 0 & 0 & 0\end{bmatrix}.$$

Reduce matrices σ_{14}, σ_{15}, σ_{16}, σ_{24}, σ_{25}, σ_{26}, σ_{34}, σ_{35} and σ_{36} by matrices σ_{12}, σ_{13} and σ_{23}, and obtain

$$\sigma_{14}\begin{bmatrix}1&0&0&0&0&0&0&0&0\\0&1&0&1&0&1&0&0&0\\0&0&1&1&0&0&0&0&0\end{bmatrix},$$

$$\sigma_{15}\begin{bmatrix}1&0&0&0&1&0&0&0&0\\0&1&0&0&0&0&0&0&0\\0&0&1&1&1&0&0&0&0\end{bmatrix},$$

$$\sigma_{16}\begin{bmatrix}1&0&0&0&1&1&0&0&0\\0&1&0&0&0&1&0&0&0\\0&0&1&0&0&0&0&0&0\end{bmatrix},$$

$$\sigma_{24}\begin{bmatrix}1&0&0&0&0&0&0&0&0\\0&1&0&1&0&1&0&0&0\\0&0&1&1&0&0&0&0&0\end{bmatrix},$$

$$\sigma_{25}\begin{bmatrix}0&1&0&0&0&0&0&0&0\\1&0&0&0&1&0&0&0&0\\0&0&1&1&1&0&0&0&0\end{bmatrix},$$

$$\sigma_{26}\begin{bmatrix}0&1&0&0&0&1&0&0&0\\0&0&1&0&0&0&0&0&0\\1&0&0&0&1&1&0&0&0\end{bmatrix},$$

$$\sigma_{34}\begin{bmatrix}0&0&1&1&0&0&0&0&0\\0&1&0&1&0&1&0&0&0\\1&0&0&0&0&0&0&0&0\end{bmatrix},$$

$$\sigma_{35}\begin{bmatrix}0&0&1&1&1&0&0&0&0\\1&0&0&0&1&0&0&0&0\\0&1&0&0&0&0&0&0&0\end{bmatrix},$$

$$\sigma_{36}\begin{bmatrix}0&0&1&0&0&0&0&0&0\\0&1&0&0&0&1&0&0&0\\1&0&0&0&1&1&0&0&0\end{bmatrix}.$$

Further reduce the matrices σ_{14}, σ_{15}, σ_{16}, σ_{24}, σ_{25}, σ_{26}, σ_{34}, σ_{35}, σ_{36}, the latter σ_{45}, the former σ_{46} and the latter σ_{56} by the former matrix σ_{45}, the latter matrix σ_{46} and the former matrix σ_{56}, then we obtain

$$\sigma_{14}\begin{bmatrix}1&0&0&0&0&0&0&0&0\\0&1&0&0&0&0&0&0&0\\0&0&1&0&0&0&0&0&0\end{bmatrix},$$

$$\sigma_{15}\begin{bmatrix}1&0&0&0&0&0&0&0&0\\0&1&0&0&0&0&0&0&0\\0&0&1&0&0&0&0&0&0\end{bmatrix},$$

$$\sigma_{16}\begin{bmatrix}1&0&0&0&0&0&0&0&0\\0&1&0&0&0&0&0&0&0\\0&0&1&0&0&0&0&0&0\end{bmatrix},$$

$$\sigma_{24}\begin{bmatrix}1&0&0&0&0&0&0&0&0\\0&1&0&0&0&0&0&0&0\\0&0&1&0&0&0&0&0&0\end{bmatrix},$$

$$\sigma_{25}\begin{bmatrix}0&1&0&0&0&0&0&0&0\\1&0&0&0&0&0&0&0&0\\0&0&1&0&0&0&0&0&0\end{bmatrix},$$

$$\sigma_{26}\begin{bmatrix}0&1&0&0&0&0&0&0&0\\0&0&1&0&0&0&0&0&0\\1&0&0&0&0&0&0&0&0\end{bmatrix},$$

$$\sigma_{34}\begin{bmatrix}0&0&1&0&0&0&0&0&0\\0&1&0&0&0&0&0&0&0\\1&0&0&0&0&0&0&0&0\end{bmatrix},$$

$$\sigma_{35}\begin{bmatrix}0&0&1&0&0&0&0&0&0\\1&0&0&0&0&0&0&0&0\\0&1&0&0&0&0&0&0&0\end{bmatrix},$$

$$\sigma_{36}\begin{bmatrix}0&0&1&0&0&0&0&0&0\\0&1&0&0&0&0&0&0&0\\1&0&0&0&0&0&0&0&0\end{bmatrix}.$$

Note that the latter matrix σ_{45}, the former matrix σ_{46} and the latter matrix σ_{56} are empty after reduction. Reduce matrices σ_{15}, σ_{16}, σ_{24}, σ_{25}, σ_{26}, σ_{34}, σ_{35} and σ_{36} by matrix σ_{14}, and get the following results

$$\sigma_{15}\begin{bmatrix}0&1&0&0&0&0&0&0&0\\0&0&1&0&0&0&0&0&0\end{bmatrix},$$

$$\sigma_{16}\begin{bmatrix}0&1&0&0&0&0&0&0&0\\0&0&1&0&0&0&0&0&0\end{bmatrix},$$

$$\sigma_{24}\begin{bmatrix}0&1&0&0&0&0&0&0&0\\0&0&1&0&0&0&0&0&0\end{bmatrix},$$

$$\sigma_{25}\begin{bmatrix}1&0&0&0&0&0&0&0&0\\0&0&1&0&0&0&0&0&0\end{bmatrix},$$

$$\sigma_{26}\begin{bmatrix}0&0&1&0&0&0&0&0&0\\1&0&0&0&0&0&0&0&0\end{bmatrix},$$

$$\sigma_{34}\begin{bmatrix}0&1&0&0&0&0&0&0&0\\1&0&0&0&0&0&0&0&0\end{bmatrix},$$

$$\sigma_{35}\begin{bmatrix}1&0&0&0&0&0&0&0&0\\0&1&0&0&0&0&0&0&0\end{bmatrix},$$

$$\sigma_{36}\begin{bmatrix}0&1&0&0&0&0&0&0&0\\1&0&0&0&0&0&0&0&0\end{bmatrix}.$$

Reduce matrix σ_{14} by matrix σ_{15} to get $\sigma_{14}\begin{bmatrix}1&0&0&0&0&0&0&0&0\end{bmatrix}$. Also, reduce matrices σ_{25}, σ_{26}, σ_{34}, σ_{35} and σ_{36} by matrix σ_{14} to obtain

$$\sigma_{25}\begin{bmatrix}0&0&1&0&0&0&0&0&0\end{bmatrix},$$

$$\sigma_{26}\begin{bmatrix}0&0&1&0&0&0&0&0&0\end{bmatrix},$$

$$\sigma_{34}\begin{bmatrix}0&1&0&0&0&0&0&0&0\end{bmatrix},$$

$$\sigma_{35}\begin{bmatrix}0&1&0&0&0&0&0&0&0\end{bmatrix},$$

$$\sigma_{36}\begin{bmatrix}0&1&0&0&0&0&0&0&0\end{bmatrix}.$$

Reduce matrices σ_{15}, σ_{16}, σ_{24}, σ_{26}, σ_{35} and σ_{36} by matrices σ_{25} and σ_{34}, and they all become empty matrices.

Now the reduction process is finished. Except for nine empty matrices, each of the remaining matrices has only one row with only one "1" element. Therefore, this model is globally identifiable.

Benchmark Model 6. Consider the mixed graph in Fig. S-5, which has been studied by Brito and Pearl [4]. Note that there are two different edges (one directed and one undirected) from node V_3 to node V_5 and from node V_3 to node V_5, respectively.

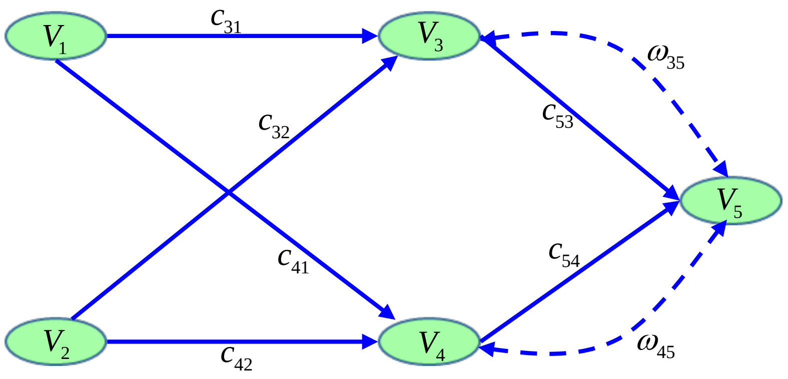

Figure S-5. A mixed graph with five nodes and repeated edges.

There are eight parameters in this model and its identifiability equations are given as follows

$$\begin{cases} \sigma_{13} = c_{31} \\ \sigma_{23} = c_{32} \\ \sigma_{14} = c_{41} \\ \sigma_{24} = c_{42} \\ \sigma_{15} = c_{31}c_{53} + c_{41}c_{54} \\ \sigma_{25} = c_{42}c_{54} + c_{32}c_{53} \\ \sigma_{35} = c_{53} + \omega_{35} + c_{31}c_{41}c_{54} + c_{32}c_{42}c_{54} \\ \sigma_{45} = c_{54} + \omega_{45} + c_{31}c_{41}c_{53} + c_{32}c_{42}c_{53} \end{cases}.$$

The columns of the identifiability matrices correspond to $\{c_{31}, c_{32}, c_{41}, c_{42}, c_{53}, c_{54}, \omega_{35}, \omega_{45}\}$, respectively, and then the identifiability matrices can be derived as follows

$\sigma_{13}\begin{bmatrix}1 & 0 & 0 & 0 & 0 & 0 & 0 & 0\end{bmatrix}$,

$\sigma_{23}\begin{bmatrix}0 & 1 & 0 & 0 & 0 & 0 & 0 & 0\end{bmatrix}$,

$\sigma_{14}\begin{bmatrix}0 & 0 & 1 & 0 & 0 & 0 & 0 & 0\end{bmatrix}$,

$$\sigma_{24}\begin{bmatrix}0 & 0 & 0 & 1 & 0 & 0 & 0 & 0\end{bmatrix},$$

$$\sigma_{15}\begin{bmatrix}1 & 0 & 0 & 0 & 1 & 0 & 0 & 0\\ 0 & 0 & 1 & 0 & 0 & 1 & 0 & 0\end{bmatrix},$$

$$\sigma_{25}\begin{bmatrix}0 & 0 & 0 & 1 & 0 & 1 & 0 & 0\\ 0 & 1 & 0 & 0 & 1 & 0 & 0 & 0\end{bmatrix},$$

$$\sigma_{35}\begin{bmatrix}0 & 0 & 0 & 0 & 1 & 0 & 0 & 0\\ 0 & 0 & 0 & 0 & 0 & 0 & 1 & 0\\ 1 & 0 & 1 & 0 & 0 & 1 & 0 & 0\\ 0 & 1 & 0 & 1 & 0 & 1 & 0 & 0\end{bmatrix},$$

$$\sigma_{45}\begin{bmatrix}0 & 0 & 0 & 0 & 0 & 1 & 0 & 0\\ 0 & 0 & 0 & 0 & 0 & 0 & 0 & 1\\ 1 & 0 & 1 & 0 & 1 & 0 & 0 & 0\\ 0 & 1 & 0 & 1 & 1 & 0 & 0 & 0\end{bmatrix}.$$

Reduce matrices σ_{15}, σ_{25}, σ_{35} and σ_{45} by matrices σ_{13}, σ_{23}, σ_{14} and σ_{24}, and get the following matrices

$$\sigma_{15}\begin{bmatrix}0 & 0 & 0 & 0 & 1 & 0 & 0 & 0\\ 0 & 0 & 0 & 0 & 0 & 1 & 0 & 0\end{bmatrix},$$

$$\sigma_{25}\begin{bmatrix}0 & 0 & 0 & 0 & 0 & 1 & 0 & 0\\ 0 & 0 & 0 & 0 & 1 & 0 & 0 & 0\end{bmatrix},$$

$$\sigma_{35}\begin{bmatrix}0 & 0 & 0 & 0 & 1 & 0 & 0 & 0\\ 0 & 0 & 0 & 0 & 0 & 0 & 1 & 0\\ 0 & 0 & 0 & 0 & 0 & 1 & 0 & 0\end{bmatrix},$$

$$\sigma_{45}\begin{bmatrix}0 & 0 & 0 & 0 & 0 & 1 & 0 & 0\\ 0 & 0 & 0 & 0 & 0 & 0 & 0 & 1\\ 0 & 0 & 0 & 0 & 1 & 0 & 0 & 0\end{bmatrix}.$$

Reduce matrices σ_{25}, σ_{35} and σ_{45} by matrix σ_{15}, and get

$$\sigma_{25}\begin{bmatrix}0 & 0 & 0 & 0 & 0 & 1 & 0 & 0\end{bmatrix},$$

$$\sigma_{35}\begin{bmatrix}0 & 0 & 0 & 0 & 0 & 0 & 1 & 0\end{bmatrix},$$

$$\sigma_{45}\begin{bmatrix}0 & 0 & 0 & 0 & 0 & 0 & 0 & 1\end{bmatrix}.$$

Reduce matrix σ_{15} by matrix σ_{25}, and get $\sigma_{15}\begin{bmatrix}0 & 0 & 0 & 0 & 1 & 0 & 0 & 0\end{bmatrix}$.

Now the reduction process is finished, and each matrix has only one row with only one "1" element. Therefore, this model is globally identifiable.

References

1. Sullivant S, Garcia-Puente LD, Spielvogel S. Identifying causal effects with computer algebra; 2010.

2. Drton M, Foygel R, Sullivant S (2011) Global identifiability of linear structural equation models. The Annals of Statistics: 865-886.

3. Kline RB (2005) Principles and Practice of Structural Equation Modeling (2nd ed.). New York: Guilford Press.

4. Brito C, Pearl J (2012) Graphical condition for identification in recursive SEM. arXiv preprint arXiv:12066821.
